# Brain‐Inspired In‐Memory Data Pruning and Computing with TaO_*x*_ Mem‐Selectors

**DOI:** 10.1002/adma.202502168

**Published:** 2025-08-25

**Authors:** Yi Li, Jinru Lai, Songqi Wang, Ning Lin, Xu Zheng, Wenxuan Sun, Danian Dong, Xiqing Xu, Haili Ma, Feng Zhang, Xiaojuan Qi, Zhongrui Wang, Xiaoxin Xu, Dashan Shang, Han Wang, Ming Liu

**Affiliations:** ^1^ Department of Electrical and Electronic Engineering The University of Hong Kong Hong Kong SAR 999077 China; ^2^ School of Microelectronics Southern University of Science and Technology Shenzhen 518055 China; ^3^ Center for Advanced Semiconductor and Integrated Circuit The University of Hong Kong Hong Kong SAR 999077 China; ^4^ State Key Lab of Fabrication Technologies for Integrated Circuits Institute of Microelectronics of the Chinese Academy of Sciences Beijing 100029 China; ^5^ School of Microelectronics University of Science and Technology of China Hefei 230026 China; ^6^ Key Laboratory of Fabrication Technologies for Integrated Circuits Chinese Academy of Sciences Beijing 100029 China; ^7^ University of Chinese Academy of Sciences Beijing 100049 China; ^8^ School of Materials Science and Engineering Chang'an University Xi'an 710061 China; ^9^ Institute of Microelectronics of the Chinese Academy of Sciences Beijing 100029 China; ^10^ Fudan University Shanghai 200433 China

**Keywords:** data pruning, in‐memory computing, memristor, neuromorphic computing

## Abstract

The selective attention mechanisms inherent in the human visual system provide a promising framework for developing edge systems that can simultaneously prune and process critical information from visual input. However, conventional complementary metal‐oxide‐semiconductor‐based edge vision systems rely on complex digital logic for data pruning, alongside the physical separation of pruning, memory, and processing. This increases both power consumption and latency. Herein, a Mem‐Selector (M‐S) device that features reconfigurable non‐volatile resistive memory and volatile threshold switching in a Ta/TaO_*x*_/Ta_2_O_5_ stack is presented. For the first time, using transmission electron microscopy, the formation and rupture of conductive oxygen vacancy filaments are observed when the device operates as a resistive memory, as well as the growth of Ta‐rich nanocrystalline clusters when it switches to threshold mode. This suggests the coexistence of ionic and electronic switching mechanisms. By leveraging a multifunctional M‐S device, an in‐memory pruning‐computing (IMPC) system that simultaneously prunes and processes information is constructed. The IMPC system, inspired by human visual‐selective attention, the IMPC system adaptively extracts essential information while pruning trivial inputs based on task complexity. This approach optimizes the balance between hardware cost and classification performance. Compared to conventional in‐memory computing systems, the integrated IMPC system reduces input energy consumption by 29%, 54%, and 90% with less than 1% accuracy loss. Additionally, it shows robustness improvements of 7.6%, 29.8%, and 80.7% on the CIFAR‐10, FashionMNIST, and MNIST datasets, respectively. This demonstrates the potential of hardware‐software co‐design for energy‐efficient, high‐performance edge hardware.

## Graphical abstract

In article number 2502168, Zhongrui Wang, Xiaoxin Xu, Dashan Shang, and co‐workers present the first nanoscale Mem‐Selector device that integrates both nonvolatile resistive memory and volatile threshold switching functionalities for visual data pruning. By co‐locating pruning, storage, and computation‐analogous to biological synapses‐such device can concurrently filter and process essential information, significantly reducing data movement overhead and facilitating energy‐efficient, low‐latency edge intelligence.

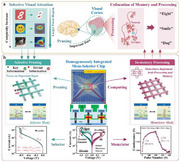

## 1 Introduction

**Figure 1:**
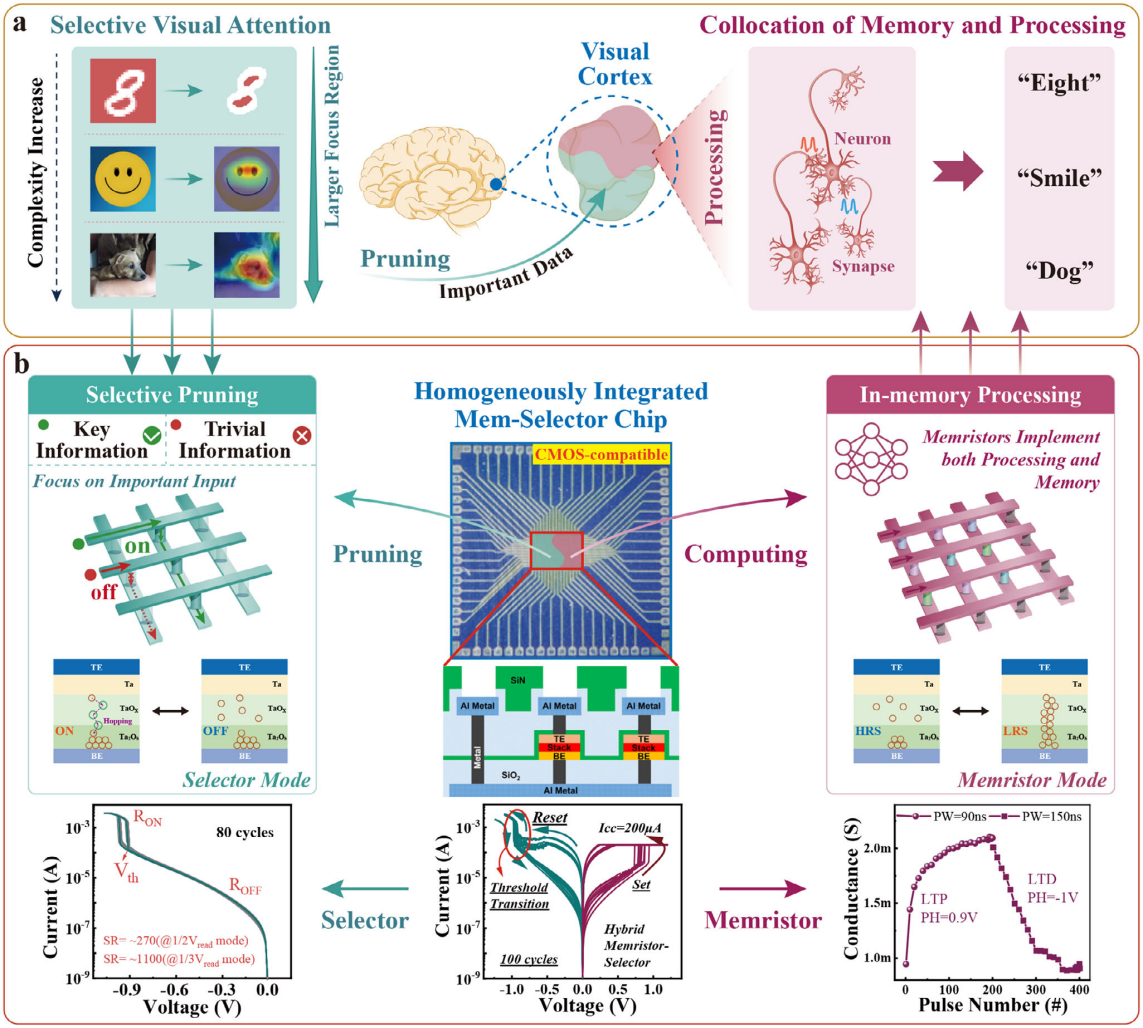
In‐memory pruning‐computing system (IMPC) with bio‐inspired integrated Mem‐Selector (M‐S) devices. a) Human visual system effectively manages tasks of varying complexity by pruning trivial information and efficiently processing key visual input. For example, when recognizing the number eight, a smiley face, or a dog, the visual cortex highlights critical regions in the image and dynamically adjusts the focus area for optimal recognition. b) Drawing inspiration from this, we developed a homogeneously integrated IMPC system using a Ta/TaO_*x*_/Ta_2_O_5_ M‐S device array (lower panel). The selector behavior of the M‐S devices enables data pruning, while their memristor behavior facilitates efficient in‐memory processing.

The human eye can process 10^8^ − 10^9^ bits of visual information per second.^[^ ^1^, ^2^ ^]^ This remarkable ability is attributed to the intrinsic visual attention mechanism, which selectively prunes visual input data, and shifts focus to informative regions via adaptable receptive fields (**Figure** **1**a). According to Kastner's theory, multiple stimuli compete for representation in the visual cortex, where stimuli with higher saliency and contrast generate a stimulus‐driven, bottom‐up bias.^[^ ^3^, ^4^ ^]^ This bias preferentially activates neurons associated with these salient stimuli, effectively pruning out less‐relevant information. Additionally, because the brain stores and processes information at synapses, there is no need to transfer massive amounts of neural network parameters, enabling the visual system to operate with extremely low energy consumption, even during high‐level cognitive tasks.

Despite the emergence of novel artificial intelligence (AI) models, such as vision transformers,^[^ ^5^ ^]^ AI hardware that emulates the brain's visual‐selective attention for data pruning and parallel brain energy efficiency through the collocation of memory and processing has yet to be demonstrated. This brain‐inspired hardware has a wide spectrum of downstream applications for autonomous driving and surveillance.^[^ ^6^ ^]^ The challenge arises because edge smart vision systems are built on complementary metal–oxide–semiconductor (CMOS) digital circuits (e.g., central and graphics processing units). First, the deceleration of Moore's law has slowed performance improvements as transistor dimensions approach their physical limits.^[^ ^7^, ^8^, ^9^, ^10^, ^11^, ^12^, ^13^, ^14^, ^15^, ^16^, ^17^, ^18^, ^19^, ^20^, ^21^, ^22^, ^23^ ^]^ Additionally, these platforms face significant data transfer challenges between off‐chip memory and processing units, resulting in high energy consumption and latency. This issue is commonly known as the von Neumann bottleneck.^[^ ^24^, ^25^, ^26^, ^27^, ^28^, ^29^, ^30^, ^31^, ^32^, ^33^ ^]^ Moreover, the use of Boolean logic gates for visual‐selective attention differs fundamentally from the analog and efficient way of the brain.^[^ ^34^, ^35^ ^]^

To address these challenges, we developed a multifunctional Mem‐Selector (M‐S) nanodevice using a TaN/Ta/TaO_*x*_/Ta_2_O_5_/TiN material stack. The device exhibits nonvolatile resistive memory (RM) under low‐voltage biasing owing to the migration of oxygen vacancies and the formation of nanoconductive filaments (CFs), as revealed by transmission electron microscopy (TEM). Leveraging the principle of “compute in physics”, the nonvolatile M‐S device physically maps neural network weights through conductance, enabling “one‐step” vector‐matrix multiplication calculations based on Ohm's and Kirchhoff's laws.^[^ ^36^, ^37^, ^38^, ^39^, ^40^ ^]^ By collocating memory and computation, the M‐S device effectively mitigates the von Neumann bottleneck, enhancing both energy efficiency and parallelism. However, when subjected to a higher voltage following the reset process, the M‐S device exhibited volatile threshold switching (TS) owing to electron hopping. Its sensitivity to voltage amplitude in this volatile state makes it well‐suited for pruning small‐voltage signals that contain negligible or no information, while preserving larger voltage signals. The reconfigurability of M‐S renders them suitable for both information pruning and in‐memory processing. Furthermore, M‐S cells are CMOS‐compatible and highly scalable due to their simple capacitor‐like structure, allowing for high integration density.

In this study, we experimentally fabricated a 32 × 32 1 Kb M‐S array integrated at the backend‐of‐line process on a standard CMOS platform. Based on this, we developed an in‐memory pruning‐computing (IMPC) system to emulate human visual attention and the collocation of memory and processing (Figure 1b). The IMPC system detects essential information in visual inputs while pruning trivial information and can adjust the ratio of neglected information according to the task complexity. This flexibility allows the system to optimize both efficiency and model performance. Compared to the in‐memory computing (IMC)‐only system, the IMPC system significantly reduces input energy consumption by 29%, 54%, and 90% on the CIFAR‐10, FashionMNIST, and MNIST datasets,^[^ ^41^, ^42^, ^43^ ^]^ respectively, with less than 1% loss in accuracy. Furthermore, the IMPC system handled noisy datasets owing to the pruning of small input fluctuations, mitigating the loss of accuracy during robustness testing by 7.6%, 29.8%, and 80.7%, compared with the IMC system. These advances demonstrate the potential of the IMPC system for balanced efficiency, highly robust, and compact edge AI hardware.

## 2 Results and Discussion

### 2.1 Physical Mechanism of the Mem‐Selector Device

**Figure 2:**
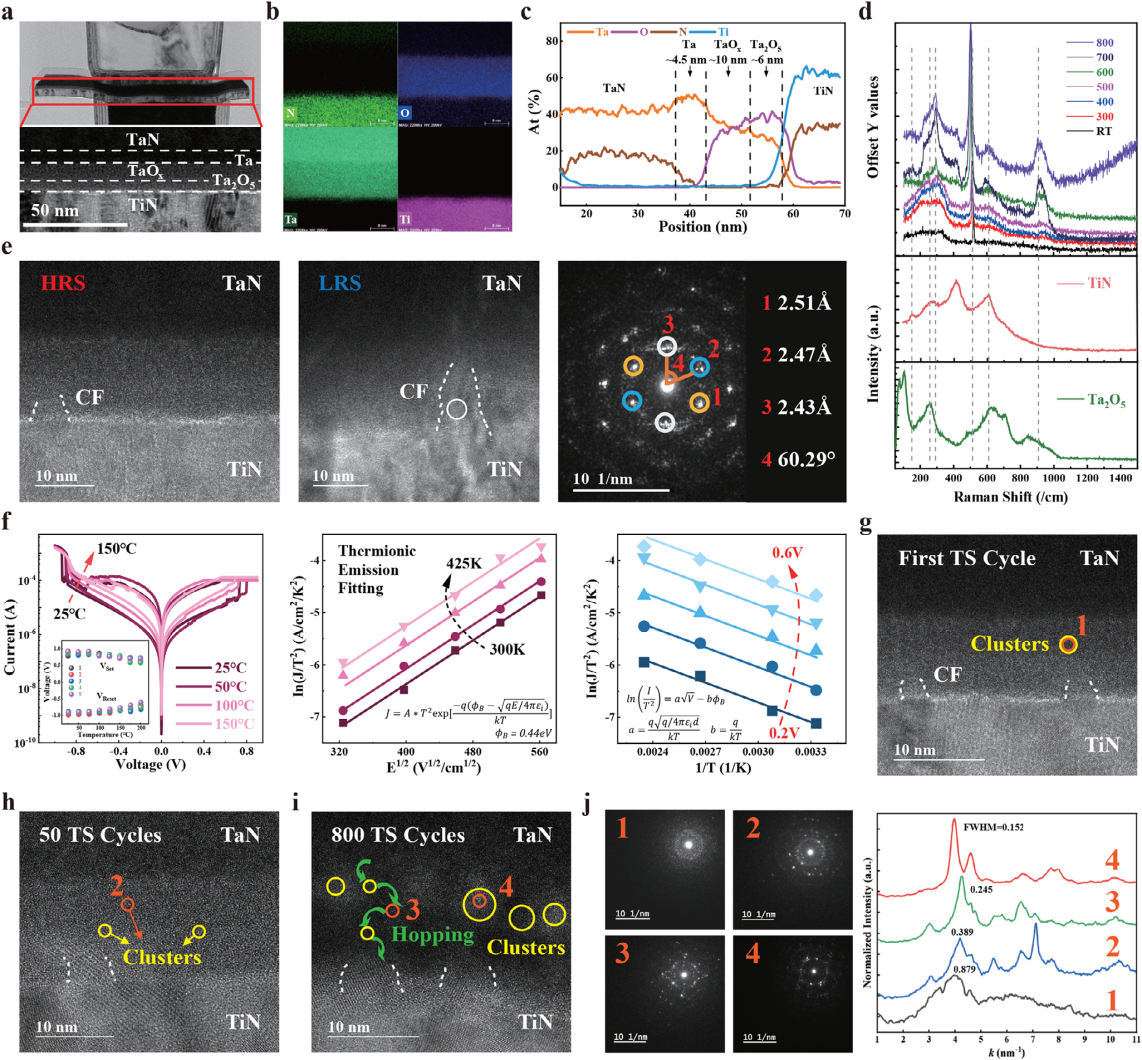
Switching and transport mechanisms of the M‐S device. a) Cross‐sectional transmission electron microscopy (TEM) image. b) Energy dispersive X‐ray spectroscopy (EDS) mapping. c) EDS line scan of the M‐S device. d) Raman spectra of the TaO_*x*_/Ta_2_O_5_/TiN film and the pure phase powders of TiN and Ta_2_O_5_. e) TEM images of the memristor mode M‐S devices in high resistance state (HRS) and low resistance state (LRS), along with the ED pattern of the white‐circled region in LRS. f) *I*–*V* sweep curves of the M‐S device at different temperatures. g) TEM images of the selector mode M‐S devices after the first threshold switching (TS) cycle. h) TEM images after 50 TS cycles. i) TEM images after 800 TS cycles. j) Nanobeam diffraction analysis and corresponding diffraction peaks' FWHM for the four positions indicated in panels (g–i).

We first probed the underlying physical mechanisms responsible for the RM behavior in the memristor mode and the TS behavior in the selector mode of the M‐S device. **Figure** **2**a presents a comprehensive transmission electron microscopy (TEM) image, along with the structural information of the M‐S device, which comprise a TaN(top electrode, TE)/Ta/TaO_*x*_/Ta_2_O_5_/TiN(bottom electrode, BE) material stack. Energy dispersive X‐ray spectroscopy (EDS) mapping (Figure 2b) and line scanning (Figure 2c) of the M‐S device revealed that the thicknesses of the Ta_2_O_5_, TaO_*x*_, and Ta layers were 6, 10, and 4.5 nm, respectively. By modulating the O_2_ and Ar flux ratios during the fabrication process, TaO_*x*_ thin films with varying stoichiometries were produced (see the X‐ray photoelectron spectroscopy results for different Ta/O ratios in Figure S1, Supporting Information). According to the X‐ray diffraction (XRD) results shown in Figure S2 (Supporting Information), both Ta_2_O_5_ and TaO_*x*_ are in an amorphous state.

To provide a more comprehensive analysis of the RM mechanisms in M‐S devices, we first investigated the impact of different substrates on the crystallization behavior of Ta_2_O_5_/TaO_*x*_ materials. Figure 2d shows the Raman spectra obtained after depositing Ta_2_O_5_ on the TiN BE and annealing it at various temperatures. A crystalline phase of Ta_2_O_5_ emerges when the annealing temperature exceeds 600 °C, as further confirmed by the X‐ray photoelectron spectroscopy results in Figure S3 (Supporting Information). In contrast, using SiO_2_ as the substrate without TiN BE does not induce the formation of a crystalline Ta_2_O_5_ phase, even at annealing temperatures of 700 °C Figure S4 (Supporting Information). Consequently, the TiN BE promotes the crystallization of Ta_2_O_5_ and accelerates the growth of CFs. Figure 2e presents the TEM images of the M‐S device in both the high resistance state (HRS) and low resistance state (LRS), along with electron diffraction (ED) patterns. During the setting process, oxygen ions in the Ta_2_O_5_ resistive layer migrated to the TaO_*x*_ layer under a positive voltage bias, forming oxygen vacancy filaments in the Ta_2_O_5_ layer.^[^ ^44^ ^]^ As shown in the TEM images, these oxygen‐deficient CFs, exhibiting a high degree of crystallinity, were initiated at the surface of the TiN BE, enhancing the device's conductivity and transitioning it from the HRS to the LRS. Conversely, when a reverse voltage was applied to reset the device, the oxygen ions stored in the TaO_*x*_ layer migrated back to the Ta_2_O_5_ layer and recombined with the oxygen vacancies in the CFs, disrupting the filaments and resetting the device to the HRS (see Figure S5, Supporting Information for a schematic of the RM process). Following the formation and disruption of CFs, a clear crystalline TaO_2_ phase was observed at the interface between Ta_2_O_5_ and TiN.

Next, we explore the TS mechanism of M‐S devices in the selector mode. Initially, we assessed the current–voltage (*I*–*V*) characteristics of the M‐S device at various temperatures. As shown in Figure 2f, the plots of Ln(J T^−2^) versus E^0.5^ and T^−1^ exhibit strong linear correlations from 300 to 425 K, fitting well with the thermionic emission mechanism.^[^ ^45^ ^]^ This yields an estimated barrier height of 0.44 eV, indicating that thermionic emission is the primary conduction mechanism in the OFF state. As the applied voltage increases, the current driven by the migration of oxygen ions from TaO_*x*_ to Ta_2_O_5_ increases, leading to increased Joule heating (Figure S6, Supporting Information). This, in turn, enhances the carrier excitation rate, creating a positive feedback loop. Once the voltage surpasses the threshold voltage (V_th_), localized Ta‐rich nanocrystalline clusters form at the TaO_*x*_/Ta_2_O_5_ interface and within the TaO_*x*_ layer. These clusters provide pathways for the rapid hopping of electrons under an electric field, enabling the transition of the device's conduction mechanism from thermionic emission to electronic hopping.^[^ ^46^ ^]^ This results in a sudden resistance drop and switches the M‐S device from the OFF state to the ON state. Conversely, in devices based solely on a Ta_2_O_5_ resistive layer, TS does not occur regardless of the Ta/O ratio, because Ta‐rich crystalline regions do not form (Figure S7, Supporting Information). Figure 2g–i display TEM images of the large‐voltage accelerated aging tests after the first TS cycle, 50 TS cycles, and 800 TS cycles, respectively. After one TS cycle, the microstructure remained consistent with that shown in Figure 2e. With an increasing number of TS cycles, both the number and size of nanocrystalline clusters increased. Figure 2j presents the results of nanobeam diffraction analysis and the corresponding full width at half maximum (FWHM) of the diffraction peaks in four selected regions across different TS cycles. The FWHMs for positions 1–4 are 0.879, 0.389, 0.245, and 0.152, respectively, indicating a progressive improvement in the crystallinity of the Ta‐rich clusters, in line with the diffraction pattern analysis. Upon removing the voltage, oxygen ions diffuse back into the TaO_*x*_ layer, causing the nano‐crystalline clusters to shrink and the device's conductivity to decrease, thereby returning the device to its initial state (see Figure S8, Supporting Information for a schematic of the TS process). In non‐accelerated aging TS tests under normal voltage, the unstable crystallization caused by the thermo‐electric coupling was reversible and did not cause significant damage to the device (see Figure S9, Supporting Information for variable temperature testing).

### 2.2 Memristor‐Behavior of the M‐S Device

**Figure 3:**
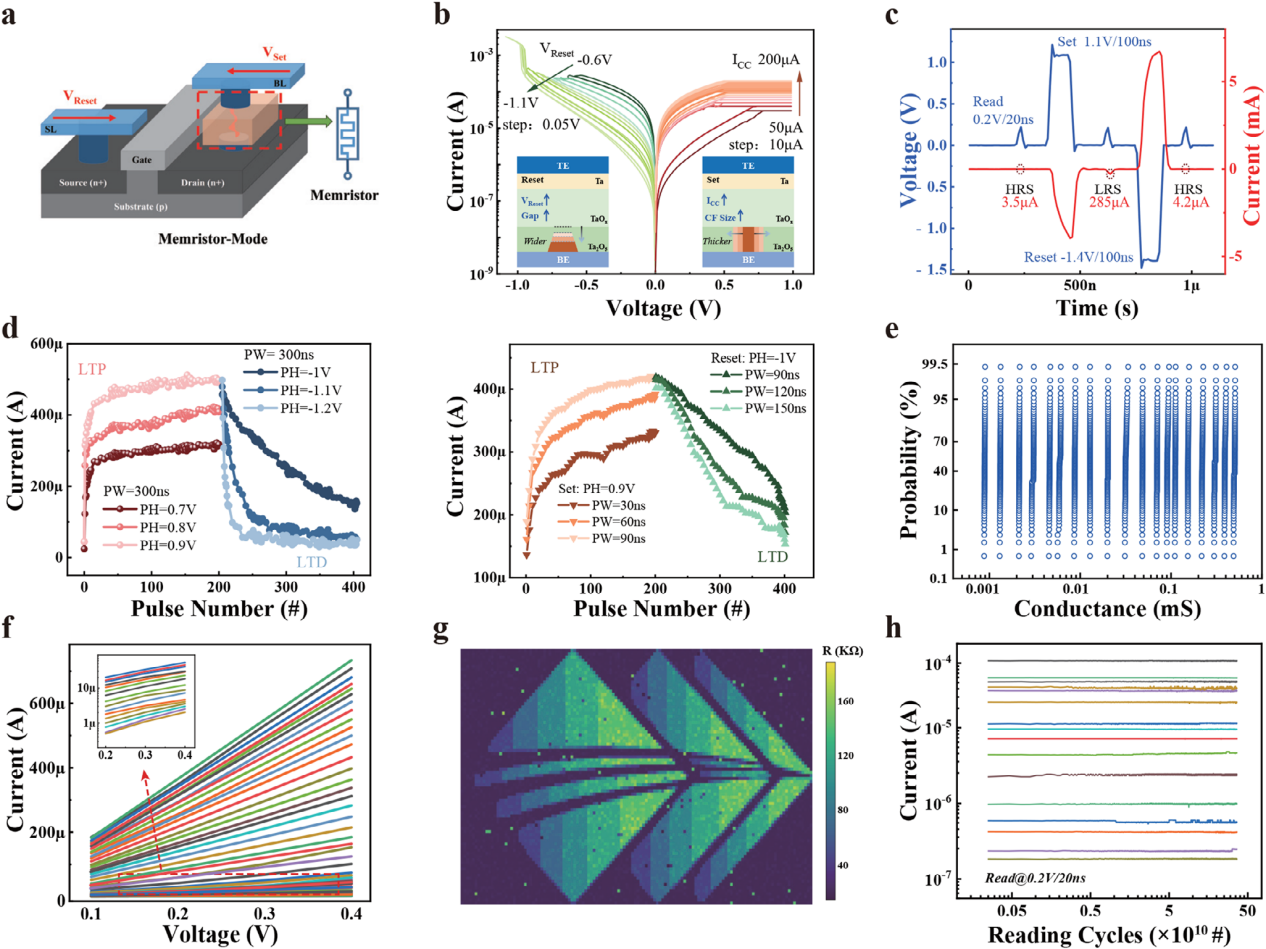
Nonvolatile memristive switching performance of the M‐S cells. a) Schematic of the M‐S device in 1‐memristor‐1‐transistor configuration. b) Quasi‐static *I*–*V* hysteresis loops of the M‐S cells in memristor mode. The device resistance is modulated by *I*_cc_ or *V*_RESET_. c) Pulsed read, set, and reset of the M‐S cells in memristor mode, demonstrating <100 ns memristive switching and <20 ns read. d) The Long‐term potentiation (LTP) and long‐term depression (LTD) plasticity using programming pulses with varying amplitudes (left) and durations (right), respectively. e) Cumulative probability distribution of 128 M‐S cells for multilevel resistance states. f) six‐bit resistance levels of the M‐S cell; the inset shows a magnified view of low conductivity levels. g) four‐bit programmed resistance heatmap of the M‐S device array showing a high yield. h) Retention test of 16 conductance states over 10^11^ reading cycles, demonstrating good stability.

Next, we present the characterization of the M‐S device operating in memristor mode. **Figure** **3**a,b present a schematic of the memristor‐mode M‐S device and the *I*–*V* characteristics measured at different compliance currents (*I*_cc_) and reset voltages (*V*_Reset_). The M‐S cells in memristor mode demonstrated fast switching speed, analog resistance states, and good reliability (Typical *I*–*V* curves of the M‐S cells are shown in Figure S10, Supporting Information).

As shown in Figure 3c, the M‐S cells exhibit a read/set/reset speed of 20/100/100 ns under a 0.2/1.1/−1.4 V voltage pulse. This corresponds to a low read energy consumption of 7.0 fJ bit^−1^ and 0.57 pJ bit^−1^ for the HRS and LRS, respectively (see Figure S11, Supporting Information for details of the M‐S read responses). The analog resistive memory of the M‐S cells emulates synaptic plasticity, including long‐term potentiation and long‐term depression, using positive and negative voltage pulses of different amplitudes and durations, as shown in Figure 3d. Figure 3e shows the cumulative probability distribution of 128 cells during multilevel resistance modulations, where all resistance states are clearly separable without overlapping. The M‐S cells achieve over 64 distinct resistance states with excellent linearity when reading across a voltage range of 0.1–0.4 V (Figure 3f), providing stable four‐bit synaptic weight resolution.

Figure 3g illustrates the high device yield in memristive programming of the M‐S device array. The four‐bit resistance map of the M‐S array revealed a clear pattern, demonstrating precise array‐level programming accuracy of 98%, due to the high device yield of the M‐S cells. Figure 3h presents the conductance retention test, where a 0.2 V reading voltage was repeatedly applied to the selected M‐S cells. Each conductance state remains stable over 10^11^ reading cycles with an average read disturbance of 1.9%, ensuring stable nonvolatile resistance for reliable analog neuromorphic computing (see Figure S14, Supporting Information for endurance tests of the M‐S devices).

### 2.3 Selector‐Behavior of the M‐S Device

We then characterized the volatile TS behavior of the M‐S cells in selector mode, demonstrating a tunable and stable threshold voltage, high endurance, and read stability. When the M‐S cells operate in selector mode, they emulate the selective visual attention of the human visual system. This process involves quickly scanning the entire image to identify the target region that requires focus, allocating more attentional resources to this region to extract detailed information, while pruning trivial data, an approach that can be implemented in the M‐S cells array.

**Figure 4:**
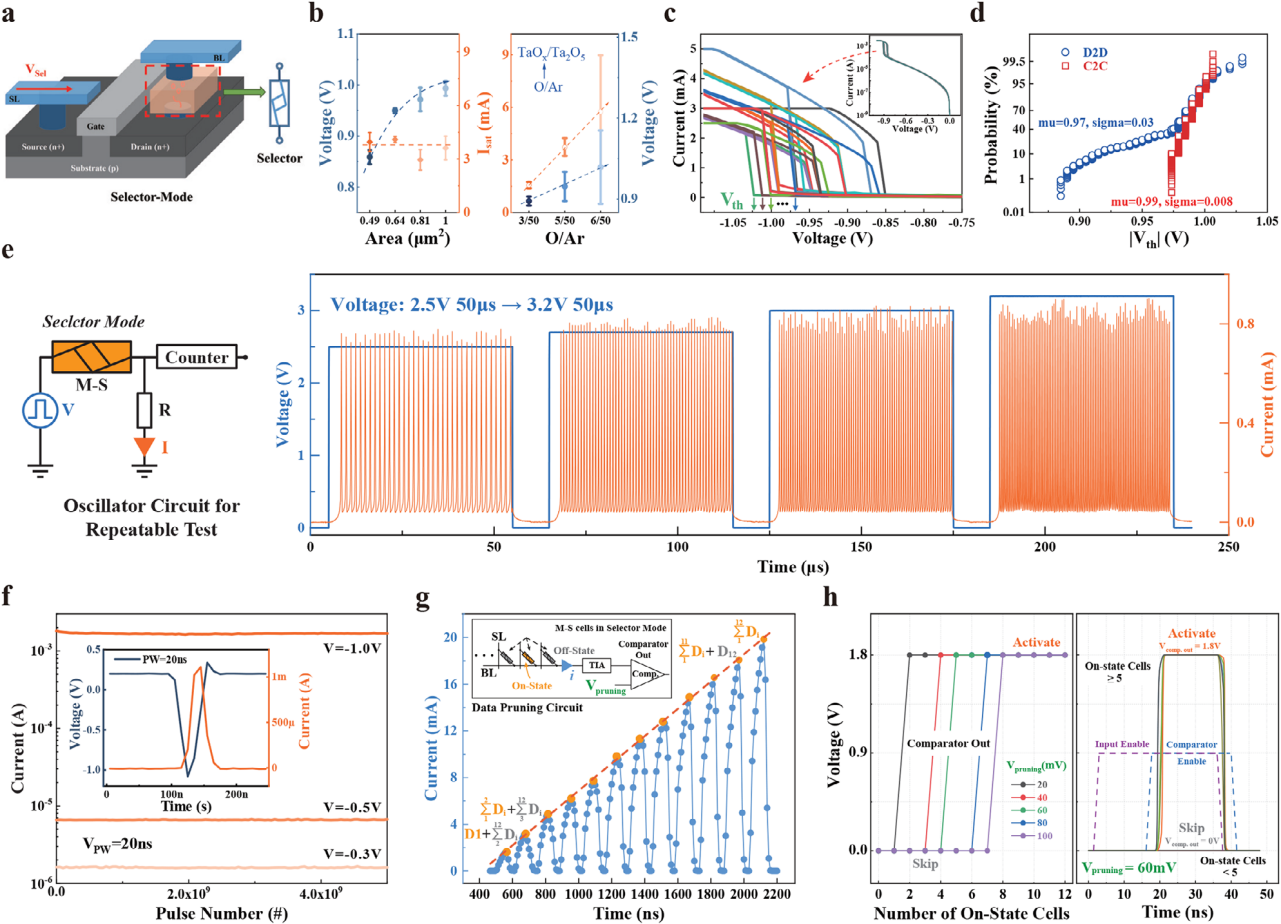
Volatile threshold switching (TS) performance of the M‐S cells. a) Schematic of the M‐S device in 1‐selector‐1‐transistor configuration. b) M‐S cell's *V*_th_ and *I*_sat_ versus cell size (left) and interfacial layer oxygen content (right). c) Measured TS characteristics of M‐S cells in selector mode. d) Cycle‐to‐cycle (C2C) and device‐to‐device (D2D) *V*_th_ distributions of the M‐S cells. e) Schematic of the oscillator circuit with the M‐S cell for repeatable testing (left) and measured spike events at a DC bias voltage in the range of 2.5 to 3.2 V (right). f) Cross‐sectional TEM images before (upper) and after 2.7 × 10^10^ cycles (lower). g) Read stability of the M‐S cells under read pulse amplitudes of *V*_th_, 1/2 *V*_th_, and 1/3 *V*_th_, each with a pulse width of 20 ns. h) Pruning circuit BL current increases with the number of M‐S cells in the ON state. The inset depicts the pruning circuit. i) Comparator output versus the number of ON state M‐S cells under different *V*_pruning_ (left) and the experimental waveform of the pruning module at *V*_pruning_ equal to 40 mV (right).

As shown in **Figure** **4**a, after resetting the M‐S device to the HRS, applying a larger voltage to the SL switches the M‐S device into the selector mode. The M‐S device demonstrated stable reconfigurability and could seamlessly switch between the two operating modes without significantly impacting each other (see Figure S17, Supporting Information for reconfigurable tests). By modifying the TaO_*x*_ layer's oxygen content during fabrication, the M‐S cell demonstrated *V*_th_ tunability. Figure 4b illustrates the correlation between *V*_th_ and the saturation current (*I*_sat_) with varying cell sizes (left) and the oxygen content of the TaO_*x*_ layer (right). As the cell size increases, the nanocrystalline regions in the TaO_*x*_ layer become more discrete, which may contribute to the increased *V*_th_ while *I*_sat_ remains almost constant. In contrast, both *V*_th_ and *I*_sat_ increased with the oxygen content in the TaO_*x*_ layer (see Figures S12, and 13 for I‐V curves under different oxygen contents and cell areas). Figure 4c shows that the *V*_th_ of the M‐S cells (1 µ*m* ^2^, O/Ar = 5/50) ranges from −0.95 to −1.05 V, indicating excellent uniformity and highly reproducible threshold switching. This is further supported by the narrow device‐to‐device and cycle‐to‐cycle |*V*_th_| distributions in Figure 4d. The measured mean and standard deviation of the |*V*_th_| distribution across 128 M‐S cells are 0.97 and 0.03 V, respectively. After 300 operational cycles, the standard deviation of the cycle‐to‐cycle |*V*_th_| distribution was 0.008 V (see Figure S16, Supporting Information for *V*_th_ drift during 10^7^ pulse tests).

To evaluate the TS repeatability of the M‐S cell, we employed the oscillator method^[^ ^47^ ^]^ which autonomously identifies cell failure. As shown in the left panel of Figure 4e, the oscillator circuit consists of a voltage supplier, an M‐S cell operating in selector mode, a load resistor, and a counter unit. During the test, a gradually increasing excitation voltage was applied to the M‐S cell to trigger TS, with higher input voltages resulting in higher oscillation frequencies. The right panel of Figure 4e presents the measured oscillator waveforms at input voltages from 2.5 to 3.2 V with a pulse width of 50 µs. The oscillator circuit exhibited uniform and stable oscillations, indicating excellent repeatability of the M‐S cell. Figure S15 (Supporting Information) demonstrates that the constructed oscillator can operate for over 10 h at 750 KHz, corresponding to an endurance of up to 2.7 × 10^10^ cycles. Compared to its initial state, the microstructure of M‐S device shows no significant defects after 2.7 × 10^10^ TS cycles, except for the formation of a small number of nanocrystalline clusters (Figure 4f). This indicates that the device possesses outstanding endurance and ensures effective, repeatable responses to input signals over extended periods. In addition to their solid TS characteristics, the M‐S cell exhibited exceptional read stability. As shown in Figure 4g, we tested the read disturbance of M‐S cells under identical pulses with amplitudes of *V*_th_, 1/2 *V*_th_, and 1/3 *V*_th_, each with a 20 ns width (see Figure S11, Supporting Information of reproducibility TS response tests). The M‐S cells could withstand more than 4 × 10^9^ pulse cycles, demonstrating a robust read performance.

Leveraging their TS characteristics, the grouped M‐S cells are naturally suited for building pruning circuits to efficiently filter input signals with small voltage amplitudes. As illustrated in the inset of Figure 4h, the pruning circuit consists of a selector mode M‐S array, a trans‐impedance amplifier (TIA), and a comparator. During data pruning, if the input voltage exceeds the M‐S cell's *V*_th_, the cell switches from the OFF to the ON state, causing a significant increase in current (***i***) through the bit line (BL). Figure 4h shows that the measured current (***i***) increased linearly with the number of M‐S cells in the ON state. This current is then converted into a voltage by the TIA and compared with a predefined threshold voltage, *V*_pruning_. When the input signals contain mostly trivial information, only a few M‐S cells are in the ON state, resulting in a converted voltage that does not reach *V*_pruning_. In this case, the comparator's output remains zero, indicating a “prune” signal. Conversely, when the input signals contain mostly important information and many cells reach *V*_th_, most M‐S cells are activated, generating a TIA output voltage greater than *V*_pruning_. This triggers the comparator to switch its output, producing an “activate” signal. Figure 4i (left) shows the comparator output versus the number of ON state M‐S cells. By adjusting *V*_pruning_, the number of ON‐state M‐S cells required to switch the comparator output can be controlled. Therefore, with proper *V*_pruning_, the pruning circuits can selectively distinguish between critical and trivial information, pruning the latter and realizing a trade‐off between system efficiency and accuracy. Figure 4i (right) shows the experimental waveform when *V*_pruning_ was set to 60 mV, with the screening results obtained within 40 ns.

### 2.4 Image Classification with the M‐S‐based Screening‐Computing System

We then developed a M‐S‐based, homogeneously integrated IMPC system by exploiting the M‐S array in the selector mode as a selective pruning module and the M‐S array in the memristor mode as an in‐memory computing module. To validate the system's capability for emulating selective visual attention, we implemented a complexity‐adaptive convolutional neural network (CNN) to classify images from the CIFAR‐10, FashionMNIST, and MNIST datasets. To demonstrate the generality of the system, we also performed audio classification tests (see Figure S25, Supporting Information for Spoken Digit classification).

**Figure 5:**
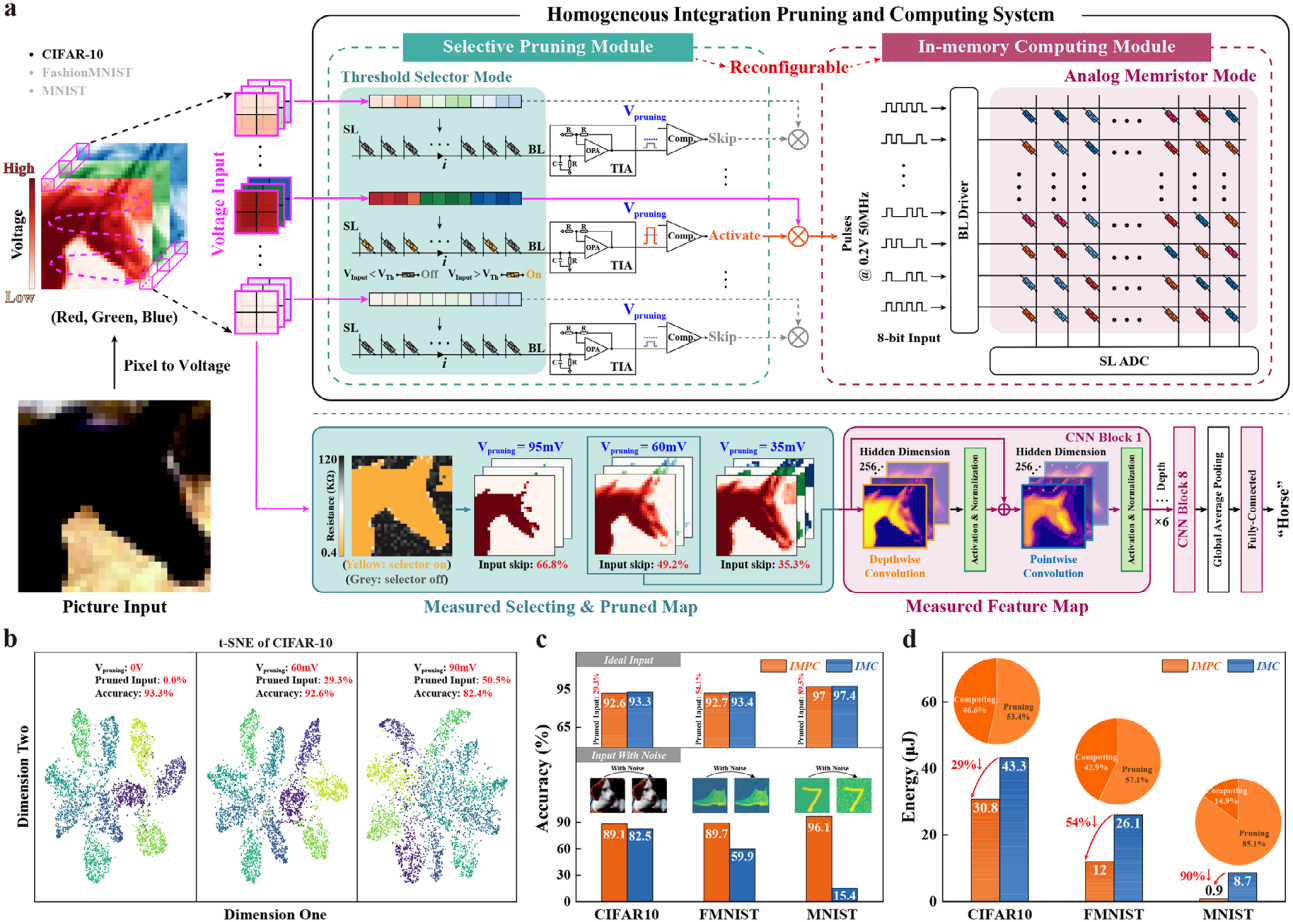
Image classification with the M‐S‐based IMPC system. a) Schematic and measured results of the image classification task implemented by the homogeneously integrated IMPC system using the developed M‐S array. During classification, test images are converted into voltage inputs and applied to the threshold selector array to prune trivial image patches. Only the activated patches are then fed into the analog memristor array for downstream classification using a convolutional neural network (CNN). b) t‐SNE plot of feature distributions on the CIFAR‐10 dataset for different *V*_pruning_ values, with data points color‐coded by category. Increasing *V*_pruning_ results in more pruned input patches, improving efficiency but reducing classification performance and leading to less distinct t‐SNE boundaries. c) Comparison of classification accuracy and robustness between the IMPC and in‐memory computing (IMC) systems. d) Comparison between IMPC and IMC input layer inference energy. The inset shows the proportion of energy consumed by the pruning and computing modules.

**Figure** **5**a presents the schematic and experimental results of the CIFAR‐10 classification. During the classification process, test images were first divided into 2 × 2 patches, converted into voltages, and applied to the selector mode M‐S array. When the input voltage (*V*_*input*_) exceeds the threshold voltage (*V*_th_), a significant increase in the bit line current (*I*_*bl*_) is observed, along with a corresponding increase in the output voltage (*V*_*convert*_), as converted by the TIA. If *V*_*convert*_ exceeds the predefined pruning voltage (*V*_pruning_), the output of the comparator flips to generate an activation signal. Conversely, patches with small voltage signals containing trivial information do not turn ON the selector or flip the comparator. By appropriately setting the pruning voltage, the dynamic pruning module adaptively identifies critical information while disregarding trivial information in different scenes, and emulates the selective visual attention of the human visual system for data pruning.^[^ ^48^ ^]^ For simple tasks, such as MNIST digit recognition, less information is required for classification; therefore, a higher *V*_pruning_ of 60 mV per input channel is used to prune input data, enhancing system efficiency. In contrast, for more complex tasks like CIFAR‐10 classification, the *V*_pruning_ is reduced to 20 mV per input channel, which decreases the pruning rate and preserves more information (see Figure S24, Supporting Information for *V*_pruning_ selection method). This adaptive mechanism effectively balances hardware cost and classification performance across different task complexities. The activated patches were then processed by a hardware‐implemented CNN using the M‐S crossbar array in memristor mode (see Figure S20, Supporting Information for system details).

Figure 5b shows the t‐distributed stochastic neighbor embedding (t‐SNE) of the CIFAR‐10 features across varying *V*_pruning_, with each point color‐coded to represent a specific category. As *V*_pruning_ increases, more input patches are pruned, leading to improved system efficiency but reduced classification performance, resulting in less clearly segregated feature clusters. Specifically, when *V*_pruning_ is set to 60 mV, the model prunes 29.3% of the input with only a 0.7% accuracy loss, effectively balancing system efficiency and performance (see Figure S22, Supporting Information for t‐SNE on the MNIST and FashionMNIST datasets).

Figure 5c compares the classification accuracy and robustness of the proposed IMPC with the conventional IMC system, which does not utilize (see Figure S21 (Supporting Information) for the RRAM‐based IMC system used in the physical chip‐level comparison). Compared to the IMC system, the IMPC system pruned 29.3%, 54.1%, and 89.5% of the input patches on the CIFAR‐10, FashionMNIST, and MNIST datasets, respectively, with a marginal accuracy loss of less than 1%. This indicates that the IMPC system can extract important information from images and maintain comparable performance. Additionally, the IMPC system excels at handling noisy samples, as the pruning mechanism filters out minor input variations. This capability prevented accuracy losses of 7.6%, 29.8%, and 80.7% during robustness testing on the respective datasets (see Figure S23, Supporting Information for an accuracy comparison at different noise levels on the three datasets).

Figure 5d compares the input energy consumption of the IMPC and IMC systems during inference. By pruning irrelevant input data, the IMPC system significantly reduces energy consumption, achieving reductions of 29%, 54%, and 90% on the three datasets, respectively, compared to the IMC system. The accompanying pie charts show the proportion of energy consumed by pruning versus computing. As task complexity decreases, the CNN model simplifies, leading to a reduction in network size and, consequently, a decrease in computational energy.

## 3 Conclusion

In this study, simultaneous threshold switching and nonvolatile resistive memory were demonstrated in a Ta/TaO_*x*_/Ta_2_O_5_ nano‐device for the first time, owing to the coexistence of ionic and electronic switching. This CMOS‐compatible M‐S device seamlessly integrates both information pruning and computation within a single unit, facilitating circuit‐level demonstrations (see Table S4 (Supporting Information) for a comparison with other devices exhibiting both short‐ and long‐term resistive switching behavior). Based on this, we developed an M‐S device array‐based IMPC system for efficient image classification at the edge. Unlike existing IMC systems, the proposed M‐S system emulates the human visual selective attention mechanism, pruning trivial input signals while preserving important ones for downstream classification using a CNN on memristor‐mode M‐S device arrays. Our IMPC system paves the way for the development of edge AI hardware that balances efficiency and performance, akin to the human brain, in the post‐Moore era.

## 4 Experimental Section

### Device Fabrication

The fabrication process of the M‐S device is shown in Figure S18 (Supporting Information). Initially, a titanium nitride (TiN) bottom electrode, with a thickness of 30 nm, was deposited onto a silicon dioxide substrate via ion beam sputtering. Subsequently, functional layers comprising tantalum pentoxide (Ta_2_O_5_), tantalum oxide (TaO_*x*_), and tantalum (Ta) were deposited using physical vapor deposition (PVD), with respective thicknesses of approximately 5, 10, and 4 nm. The fabrication process was completed by depositing a 30 nm thick tantalum nitride (TaN) top electrode. Characterized by its simple capacitor structure and compatibility with CMOS technology, the M‐S device exhibits exceptional scalability, consistent with other typical 1T1R memory structures (see Figure S19, Supporting Information for scaling potential of the M‐S device).

### Device Measurement

Electrical measurements were conducted at specific temperatures using an Agilent B1500A semiconductor parameter analyzer in conjunction with a probe station. The pulse voltage was generated through the semiconductor pulse generator unit module.

### Material Characterization

Cross‐sectional TEM, EDS, and NBD were performed using a Talos system (Thermo Fisher Scientific) equipped with a Super‐X EDS system (Thermo Fisher Scientific). Prior to TEM characterization, thin lamellae were prepared using a Helios Dual‐Beam system (Thermo Fisher Scientific).

### In‐memory Pruning‐Computing System

The in‐memory screening‐computing hardware system (see Supporting Information) consists of an 180 nm M‐S chip and a system‐on‐chip, which contains advanced RISC machines (ARM) with a field‐programmable gate array (FPGA). For the screening phase, all SLs of the selector sub‐array are biased according to pixel values sourced by an eight‐channel digital‐to‐analog converter (DAC, DAC60508, Texas Instruments) with a 12‐bit resolution, while BLs were grounded. The current from the BLs, which carries the screening result, was converted to voltages via TIA (OPA4353, Texas Instruments) and then used as the input for the comparator to generate activation signals. For the computing phase, a four‐channel analog multiplexer (Mux, CD4051B, Texas Instruments) with an eight‐bit shift register (SN74HC595, Texas Instruments) applies a direct current voltage to the BLs of the analog memory sub‐array, while the selected SL is grounded and the rest of the SLs float. The analog memory sub‐array is read, and the multiplication values represented by the SLs' current were converted by ten‐bit analog‐to‐digital converters (ADC, ADS5287, Texas Instruments). These results were then sent to the Xilinx SoC for further neural network processing, such as activation and pooling.

### Multi‐Bit Vector‐Matrix Multiplication

To perform vector‐matrix multiplication, the analog input was converted into an m‐bit binary vector, where m is 4 for MNIST, 6 for FashionMNIST, and 8 for CIFAR‐10 classifications. During each multiplication step, a small fixed voltage (e.g., 0.2 V) was applied to the bit lines of the M‐S array when the input equals ‘1’, or grounded when the input equals ‘0’. The output currents from all SLs were subsequently multiplied by their corresponding significance and aggregated in the digital domain.

### Details of the CNN Model

The design was validated on three visual datasets using the ConvMixer model. The ConvMixer model employed a block‐stacking architecture, wherein each block comprises depth‐wise and point‐wise convolution layers that mix features across spatial and channel dimensions, followed by activation functions and batch normalization. By integrating the strengths of CNN and Transformer models, the ConvMixer excels in image classification with a small parameter size. During classification, the ConvMixer model was dynamically scaled based on the proportion of pruned inputs relative to the total inputs (Table S2, Supporting Information). When the proportion of pruned inputs exceeds 85%, indicating sparse valid information across the image, a small‐sized network with a depth of two and a hidden dimension of 128 suffices to achieve high classification accuracy. Conversely, when the proportion of pruned inputs was less than 35%, suggesting a more complex image, a larger network with a depth of eight and a hidden dimension of 256 was necessary to ensure accurate classification. For intermediate proportions of pruned inputs, a medium‐sized network with a depth of four and a hidden dimension of 128 was employed. The kernel size for the depth‐wise convolution layers was set to 5 × 5. Given the model's repeating block structure and the limited chip capacity, the weights of the first block were mapped onto the M‐S chips, while the weights of the remaining blocks were sampled using the measured M‐S conductance distribution and implemented in software. The final readout layer was a fully connected network with a 256‐D hidden layer (128 for the MNIST dataset).

### System‐Level Benchmark

When benchmarking the IMPC and IMC systems' energy consumption, both the core array and peripheral circuits were considered, and the calculations were based on the experimentally measured M‐S device characteristics shown in Figures 3 and 4. For the in‐memory computing module, the energy consumption is calculated as follows:

(1) $$Array=E_{Memristor\_Mode}\times Weight\_Size\times Input\_Bits$$

where $E_{Memristor\_Mode}$, Weight_Size, and Input_Bits, *k*_*w*_ refer to the operation energy of the M‐S cell in memristor mode, the number of multiplications, and input bit width, respectively. The Weight_Size was determined by each layer of the network:

(2) $$Weight\_Size=\left(1-Input\_Pruned\right)\times out\times in\times k_{h}\times k_{w}\times height\times width,$$

where Input_Pruned, *out*, *in*, *k*_*h*_, *k*_*w*_, *height*, and *width* denote the proportion of pruned input, the convolutional layer's output channels, input channels, kernel's height and width, and the output feature maps' height and width, respectively. For the IMPC system, the *Input*_*P*_ *runed* was determined based on the task complexity, while for the IMC system, it was zero since the inputs were not pruned. The peripheral circuits were evaluated as follows:

(3) $$Drivers=E_{Driver}\times w_{h}\times w_{w}\times Input\_Bits$$

(4) $$ADC=E_{ADC}\times w_{w}\times Input\_Bits$$

(5) $$Shift\&Adder=E_{Shift\&Adder}\times w_{w}\times \left(Input\_Bits-1\right)$$

(6) $$Mux\&Decoder=E_{Mux}\times w_{w}\times Input\_Bits+E_{Decoder}\times w_{w}\times 2,$$

where *E*_*Driver*_, *E*_*ADC*_, $E_{Shift\&Adder}$, *E*_*Mux*_, *E*_*Decoder*_, *w*_*h*_, and *w*_*w*_ refer to the energy consumption of the driver, ADC, shift adder, multiplexer, and decoder for a single operation, and the height and width of the weight matrix, respectively. The total energy consumption of the in‐memory computing module was estimated as follows:

(7) Total=Array+2×(Drivers+Mux&Decoder+ADC+Shift&Adder)

where 2 × is associated with differential M‐S arrays.

For the selective pruning module, the energy consumption is calculated as follows:

(8) $$Array=E_{Selector\_Mode\_On}\times Device_{on}+E_{Selector\_Mode\_Off}\times Device_{off}$$

where $E_{Selector\_Mode\_On}$ and $E_{Selector\_Mode\_Off}$ refer to the operation energy of the ON and OFF states of the M‐S cell in selector mode. The *Device*_*on*_ and *Device*_*off*_ denote the number of ON and OFF cells:

(9) $$Device_{on}=\left(1-Input\_Pruned\right)\times in\times k_{h}\times k_{w}\times height\times width$$

(10) $$Device_{off}=Input\_Pruned\times in\times k_{h}\times k_{w}\times height\times width$$

The peripheral circuits are evaluated as follows:

(11) $$DAC=E_{DAC}\times w_{w}$$

(12) $$Mux=E_{Mux}\times w_{w}$$

(13) $$Drivers=E_{Driver}\times w_{h}\times w_{w}$$

(14) $$TIA=E_{TIA}\times height\times width,$$

where *E*_*DAC*_ and *E*_*TIA*_ denote the energy consumption of the DAC and TIA a single operation. The total energy consumption of the dynamic screening module is estimated as follows:

(15) Total=Array+DAC+Mux+Drivers+TIA

The specific hardware parameters are presented in Tables S1 and S3 (Supporting Information).

## Conflict of Interest

The authors declare no conflict of interest.

## Supporting information

Supporting Information

## Data Availability

The data that support the findings of this study are available from the corresponding author upon reasonable request.
