# Supplementary material for: Brain‐Inspired In‐Memory Data Pruning and Computing with TaO x Mem‐Selectors
Source: Adv Mater. 2025 Aug 25;37(43):e02168. doi: 10.1002/adma.202502168 (PMC12574655; doi:10.1002/adma.202502168)
Supplement: Supplementary file 1 — Supporting Information [file ADMA-37-e02168-s001.pdf]

ADVANCED MATERIALS

Supporting Information

for *Adv. Mater.*, DOI 10.1002/adma.202502168

Brain-Inspired In-Memory Data Pruning and Computing with $TaO_x$ Mem-Selectors

*Yi Li, Jinru Lai, Songqi Wang, Ning Lin, Xu Zheng, Wenxuan Sun, Danian Dong, Xiqing Xu, Haili Ma, Feng Zhang, Xiaojuan Qi, Zhongrui Wang*, Xiaoxin Xu*, Dashan Shang*, Han Wang and Ming Liu*

# Supporting Information for Brain-inspired in-memory data pruning and computing with $TaO_x$ mem-selectors

Yi Li[†], Jinru Lai[†], Songqi Wang, Ning Lin, Xu Zheng, Wenxuan Sun, Danian Dong, Xiqing Xu, Haili Ma, Feng Zhang, Xiaojuan Qi, Zhongrui Wang*, Xiaoxin Xu*, Dashan Shang*, Han Wang, and Ming Liu

May 29, 2025

# S1. Supplementary Figures

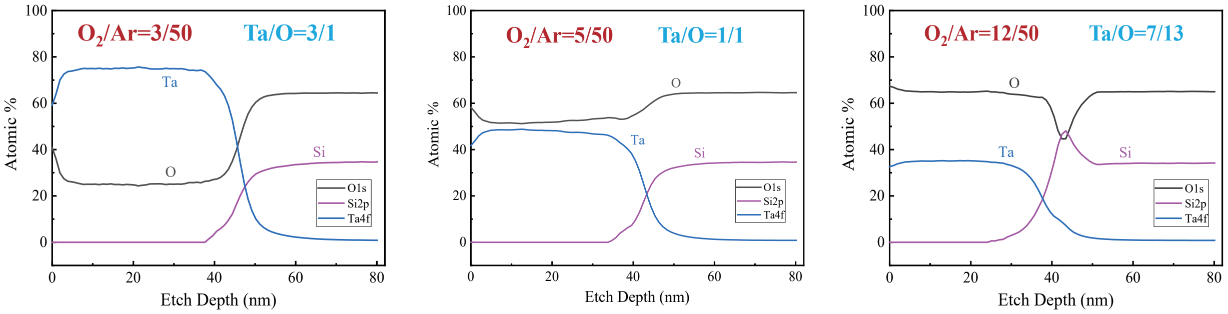


**Figure S1:** Variation in the Ta/O ratio of $TaO_x$ under different $O_2$ flux conditions. A progressive increase in oxygen content is observed with the enhancement of oxygen flux during the synthesis of $TaO_x$.

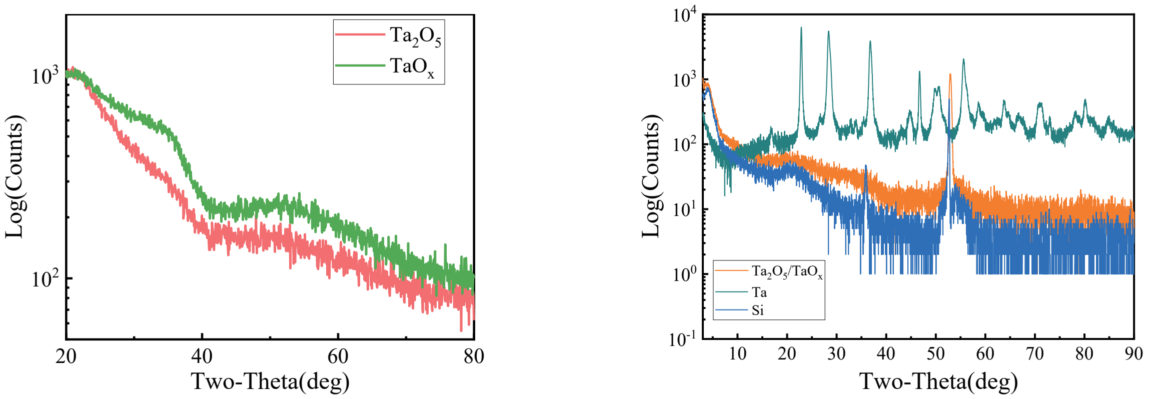


**Figure S2:** XRD analysis of $Ta_2O_5$, $TaO_x$, and $TaO_x/Ta_2O_5$ films deposited on silicon oxide substrates, indicating that all samples exhibit an amorphous phase.

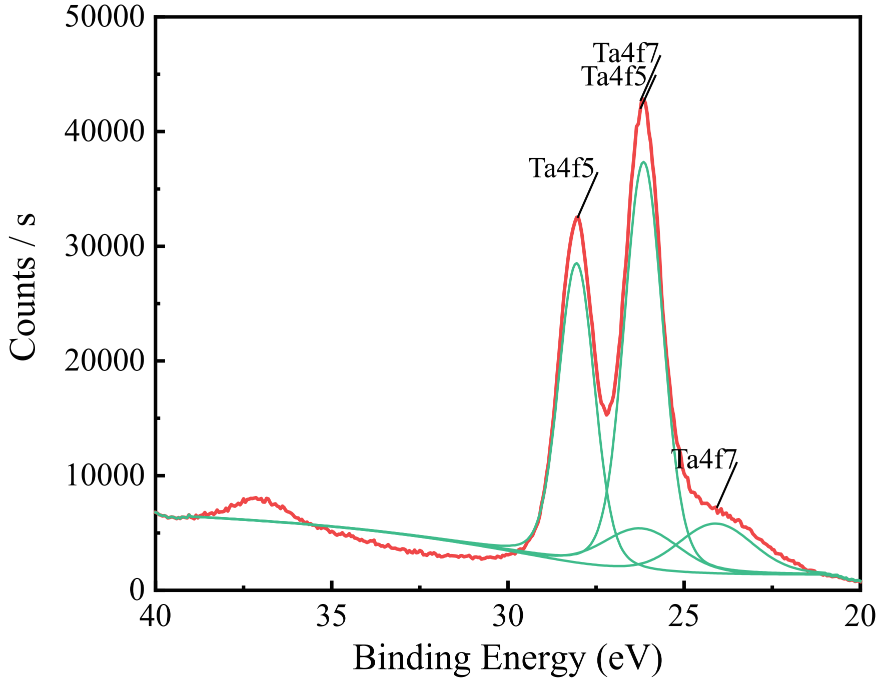


**Figure S3:** XPS result of $Ta_2O_5/TaO_x$ reveals the presence of synthesized $Ta_2O_5$ along with oxygen-deficient substoichiometric components.

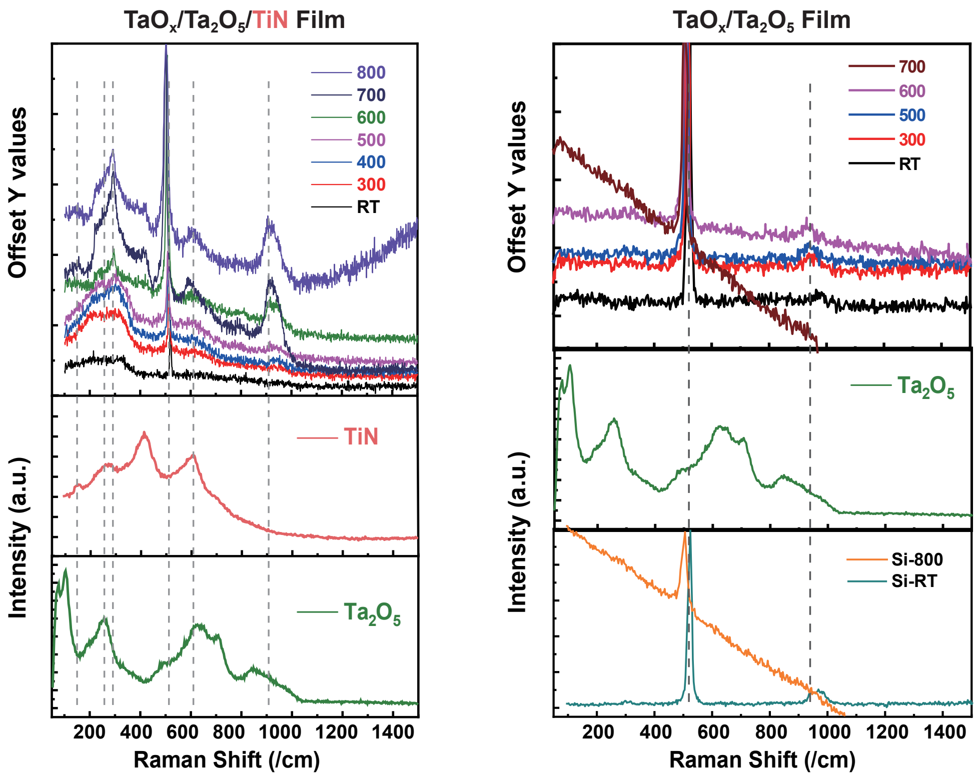


**Figure S4:** Comparison between the Raman spectra of the $TaO_x/Ta_2O_5$/TiN film and the $TaO_x/Ta_2O_5$ film at different annealing temperatures.

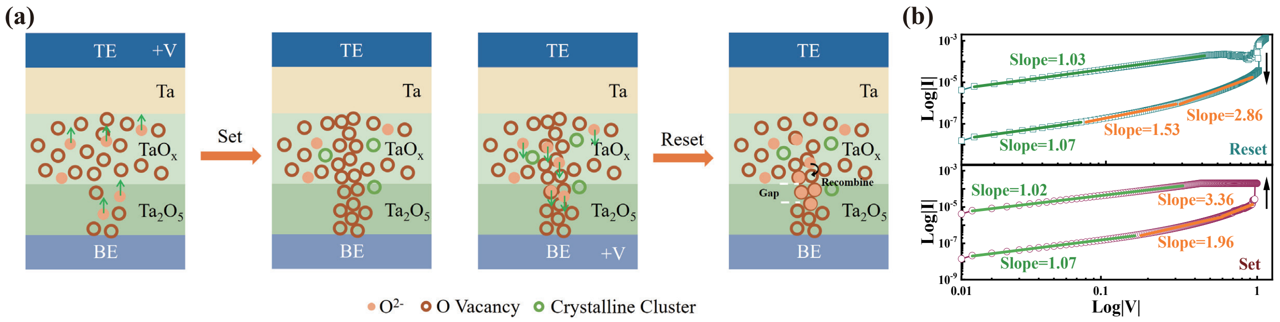


**Figure S5:** Schematic of the resistive memory (RM) process (a) and the partitioned linear fitting of the IV curves in the positive and negative bias of a double logarithmic plot (b). The conduction mechanism of the memristor mode mem-selector (M-S) device is driven by the formation and rupture of conductive filaments, attributed to the migration of oxygen vacancies. In the low resistance state (LRS), the logarithmic current shows a linear dependence on logarithmic voltage, with a slope close to 1, indicating an Ohmic conduction mechanism. [1] The high resistance state (HRS) I-V curves can be divided into distinct regions: Ohmic conduction at low fields, Child's law conduction at higher voltages, [2] and an exponential increase region during the set process, consistent with space-charge-limited conduction behavior. [3]

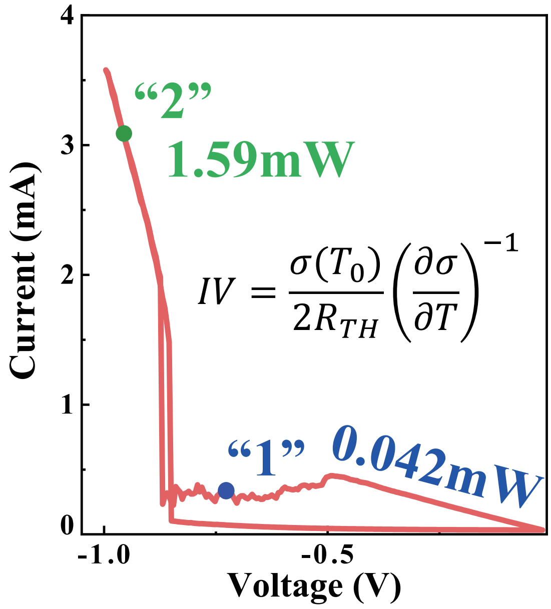


**Figure S6:** Dissipated power analysis before and after the abrupt change of current during the reset process. At the '2' point, a positive feedback loop between the current and temperature is observed, leading to a further increase in current.

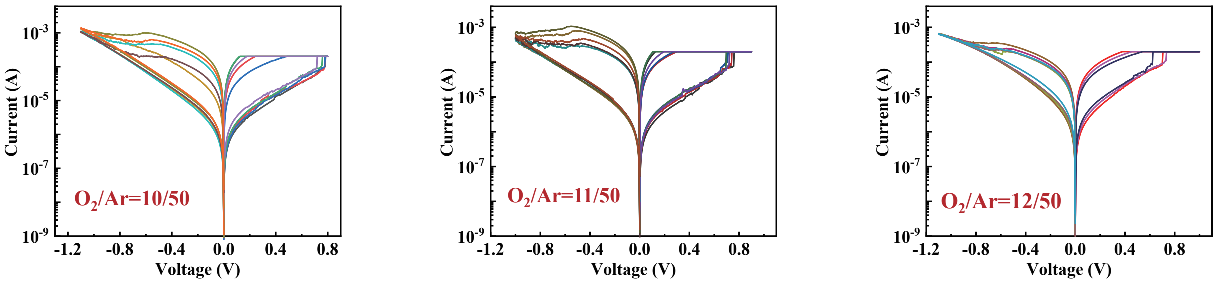


**Figure S7:** I-V characteristic curves of TiN/$Ta_2O_5$/Ta/TaN devices. Regardless of the Ta/O ratio variations, the TiN/$Ta_2O_5$/Ta/TaN devices exhibit only typical RM characteristics and do not display threshold switching (TS) characteristics.

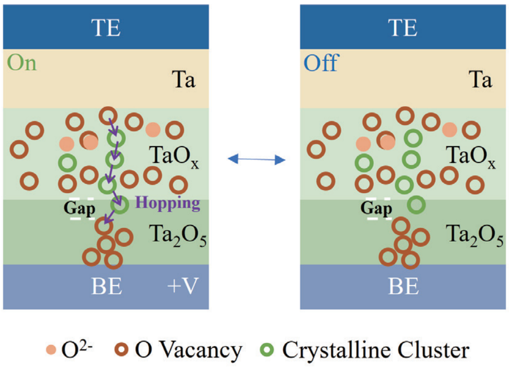


**Figure S8:** Schematic of the TS process. When a larger voltage is applied after the reset process, localized Ta-rich nano-crystalline clusters are generated at the $TaO_x/Ta_2O_5$ interface and within the $TaO_x$ layer. This causes the conduction mechanism in the M-S device to transition from thermionic emission to electron hopping, resulting in a sudden drop in resistance.

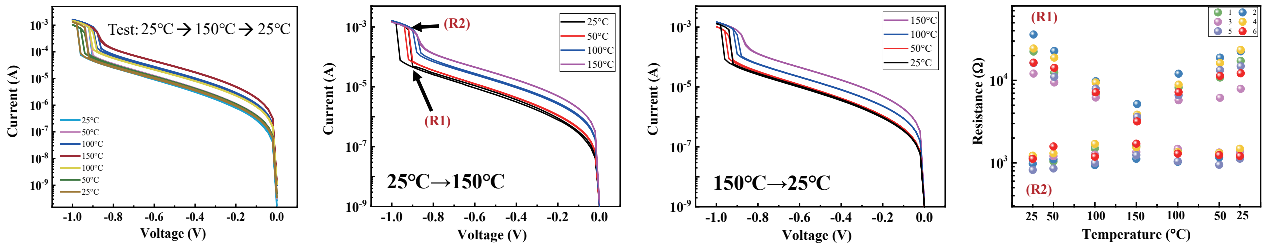


**Figure S9:** I-V curves for temperature rise and fall processes, and the trend of resistance with temperature before and after threshold switching. As the temperature increases from 25ºC to 150ºC, the leakage current of the device increases, the threshold switching voltage decreases, the selection ratio of the device decreases, and the saturation current remains constant. When the temperature decreases from 150ºC to 25ºC, the leakage current of the device decreases back to its original value, and the threshold switching voltage and selection ratio increase again, eventually returning to their initial values.

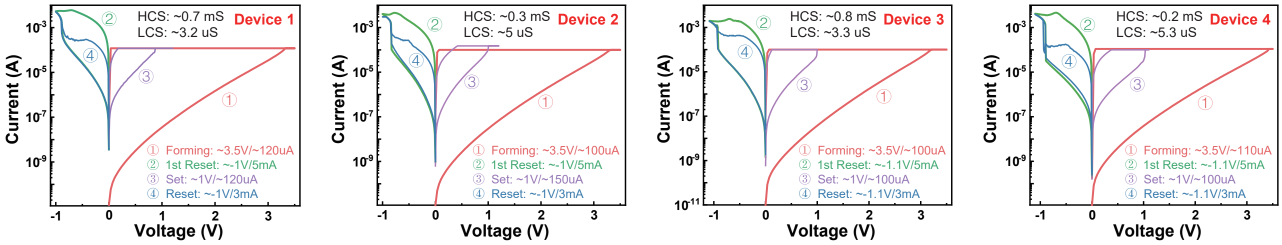


**Figure S10:** Typical I-V curves of M-S cells.

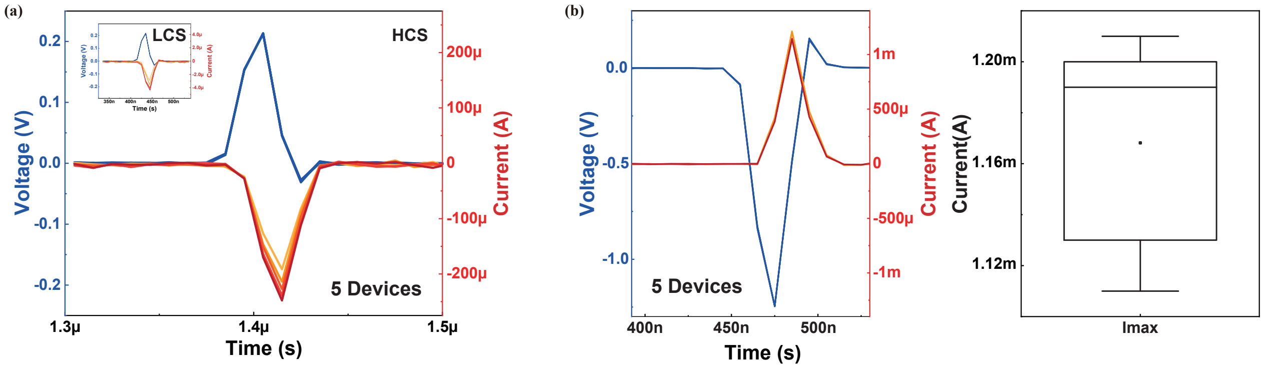


**Figure S11:** Typical M-S cell response curves. (**a**) Read response curves. (**b**) Threshold switching curves.

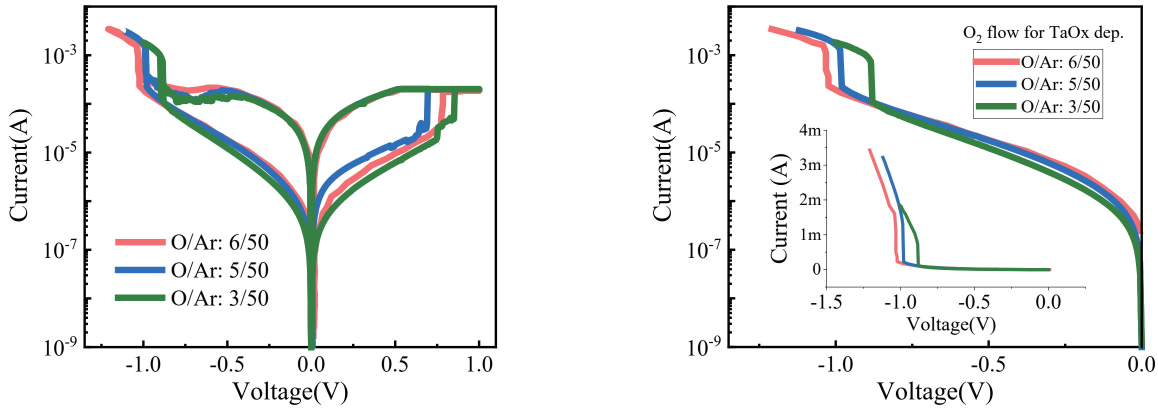


**Figure S12:** Typical I-V curves for devices with different oxygen fluxes during the deposition of $TaO_x$. As the oxygen flux increases, both the burst voltage and the saturation current also increase.

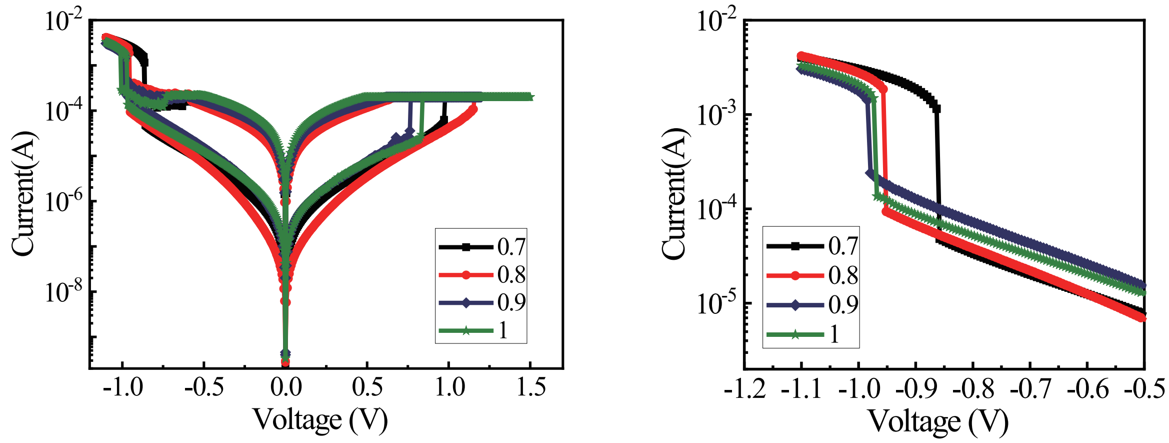


**Figure S13:** Typical I-V curves for devices with different areas show that as the device area increases, the abrupt voltage also increases, while the saturation current varies irregularly.

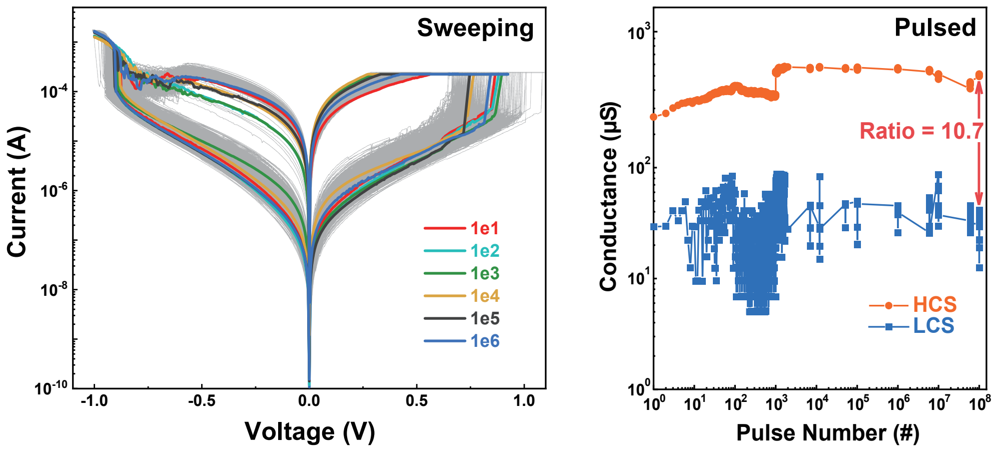


**Figure S14:** Endurance testing under sweep and pulse modes.

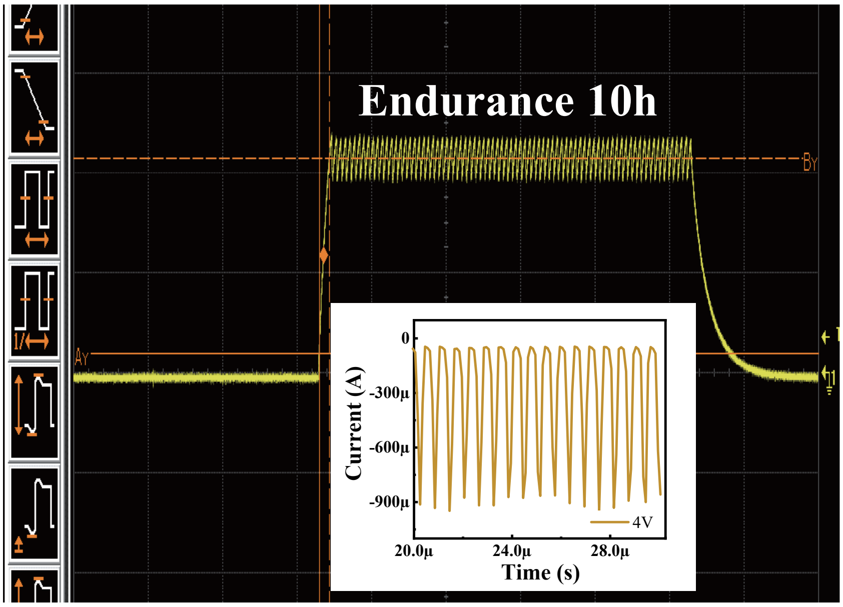


**Figure S15:** The threshold switching endurance test of the M-S cells using the oscillator method. The oscillatory waveforms depicted in the figure are plotted by sampling at fixed intervals, where the inset shows the detailed waveforms. The constructed oscillator can oscillate at a frequency of 750 kHz for more than 10 hours with a 4 V voltage input (plotting using interval recording), demonstrating durability up to 2.7 $\times 10^{10}$ cycles.

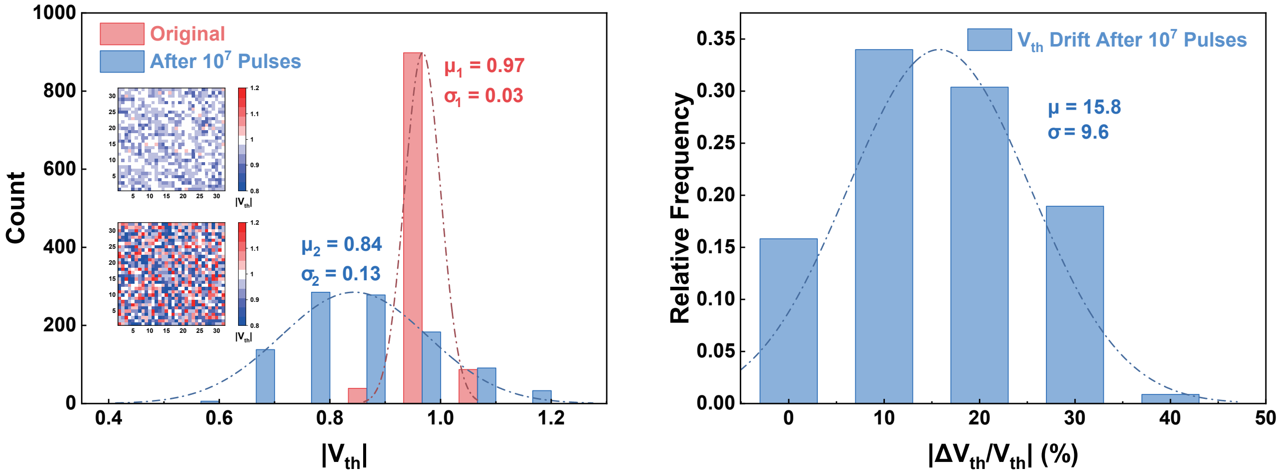


**Figure S16:** $V_{th}$ before and after $10^7$ pulse tests.

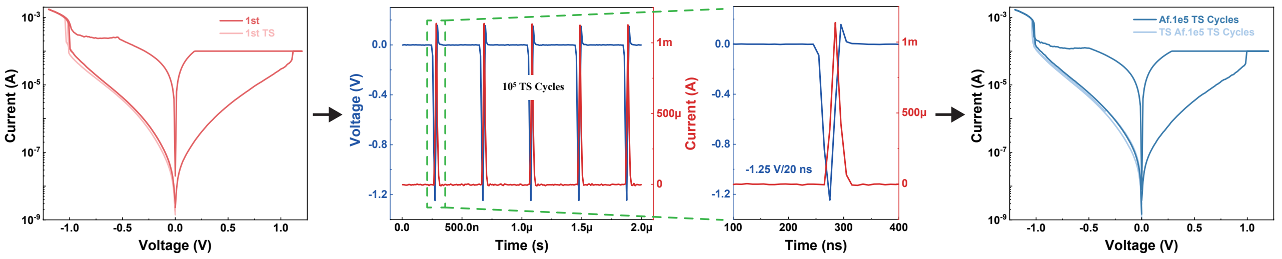


**(a)** Resistive memory characteristic after $10^5$ TS cycles.

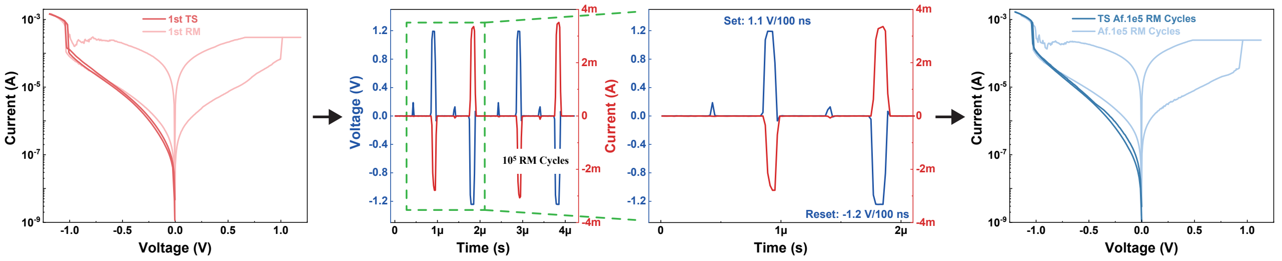


**(b)** Thresholding switching characteristic after $10^5$ RM cycles.

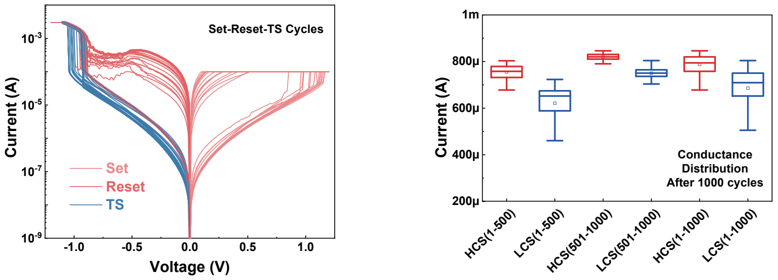


**(c)** Test results for alternative switching between memristor and selector modes.

**Figure S17:** (a) Resistive memory characteristic after $10^5$ TS cycles. (b) Thresholding switching characteristic after $10^5$ RM cycles. (c) Test results for alternative switching between memristor and selector modes.

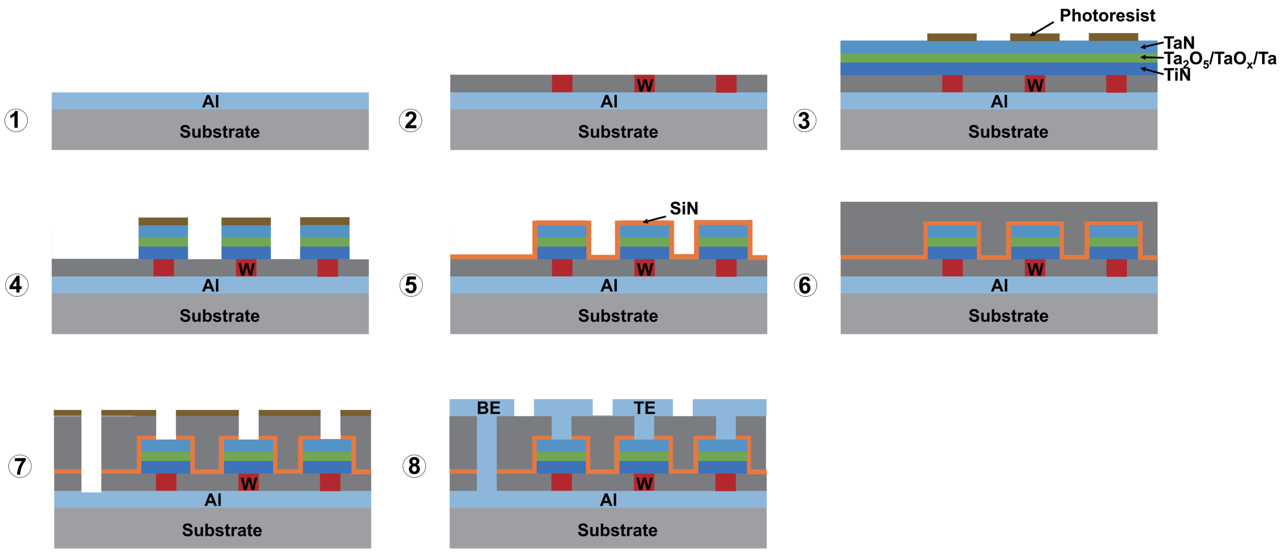


**Figure S18:** Fabrication process of the M-S device.

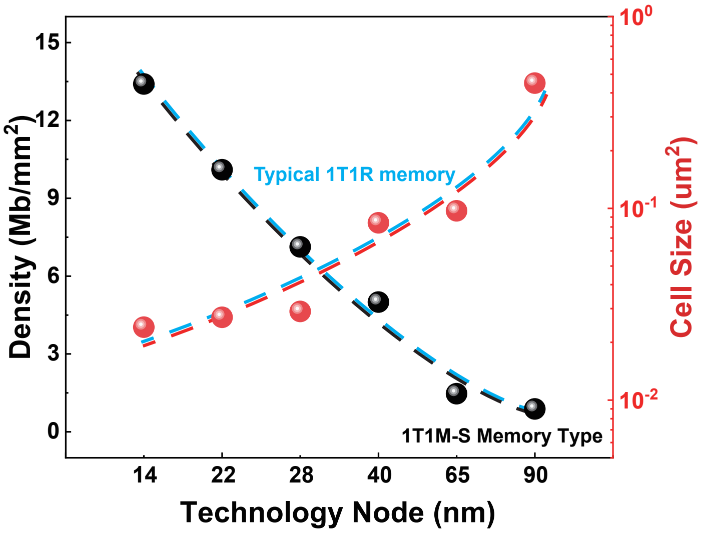


**Figure S19:** Scaling potential analysis of the M-S device.

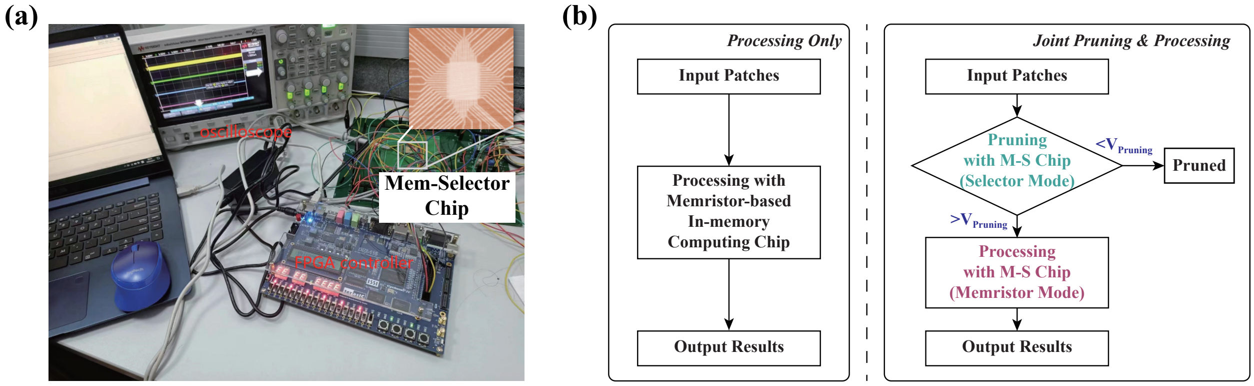


**Figure S20:** In-memory pruning-computing system. (**a**) The in-memory pruning-computing hardware system consists of an 180 nm M-S chip and a system-on-chip on a printed circuit board. (**b**) Comparison between the processing-only (left) and the joint pruning and processing (right) flowcharts.

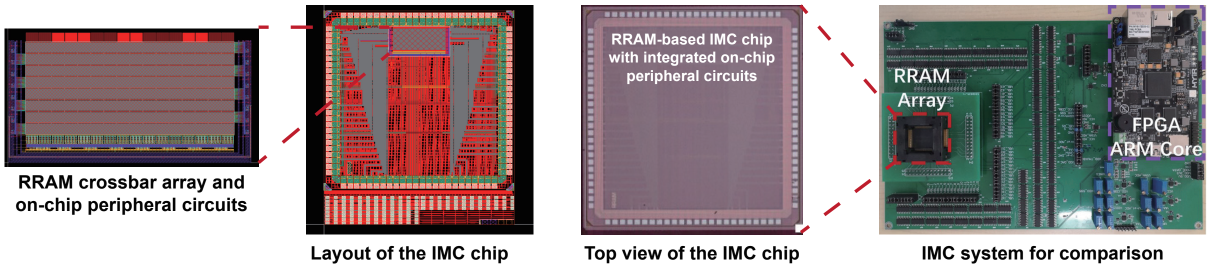


**Figure S21:** RRAM-based IMC chip with integrated on-chip peripheral circuits for physical chip-level comparison. [4]

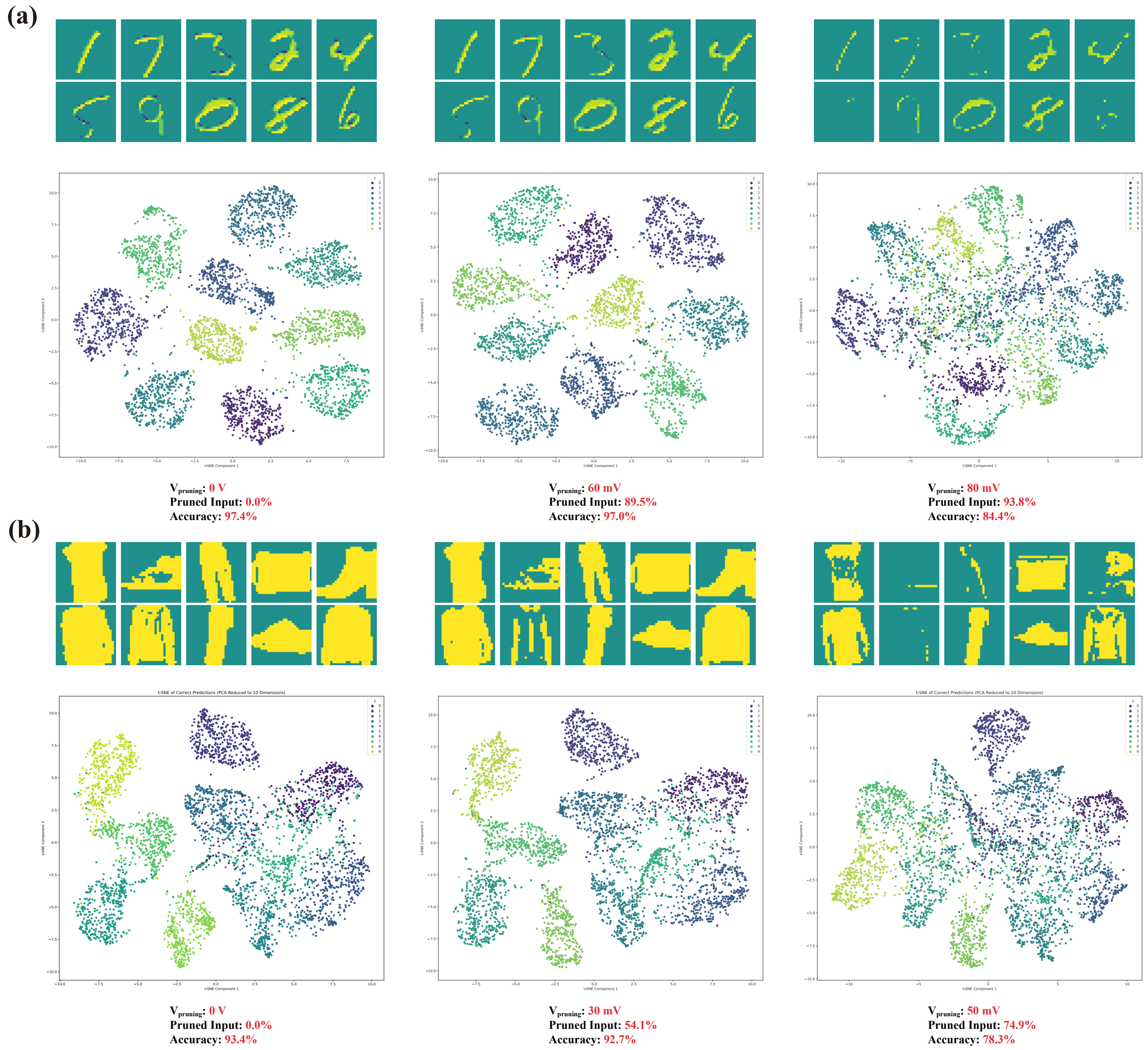


**Figure S22:** The t-SNE visualization of the MNIST (**a**) and FashionMNIST (**b**) datasets across various $V_{pruning}$ values. Notably, the model skips 89.5% and 54.1% of the inputs when $V_{pruning}$ equals 60 mV and 30 mV, respectively, with an accuracy loss of less than 1% on both datasets, indicating a successful balance between system efficiency and performance.

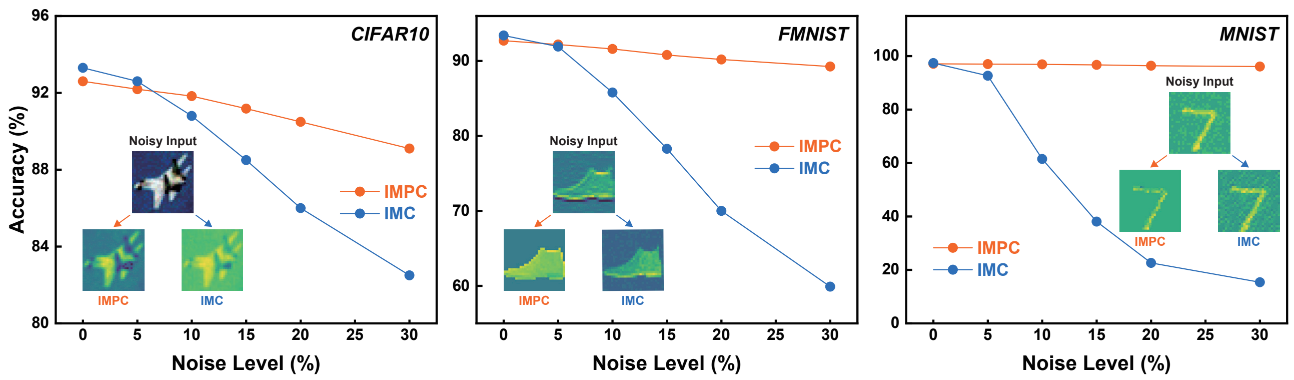


**Figure S23:** Comparison of classification accuracy at different noise levels on three datasets.

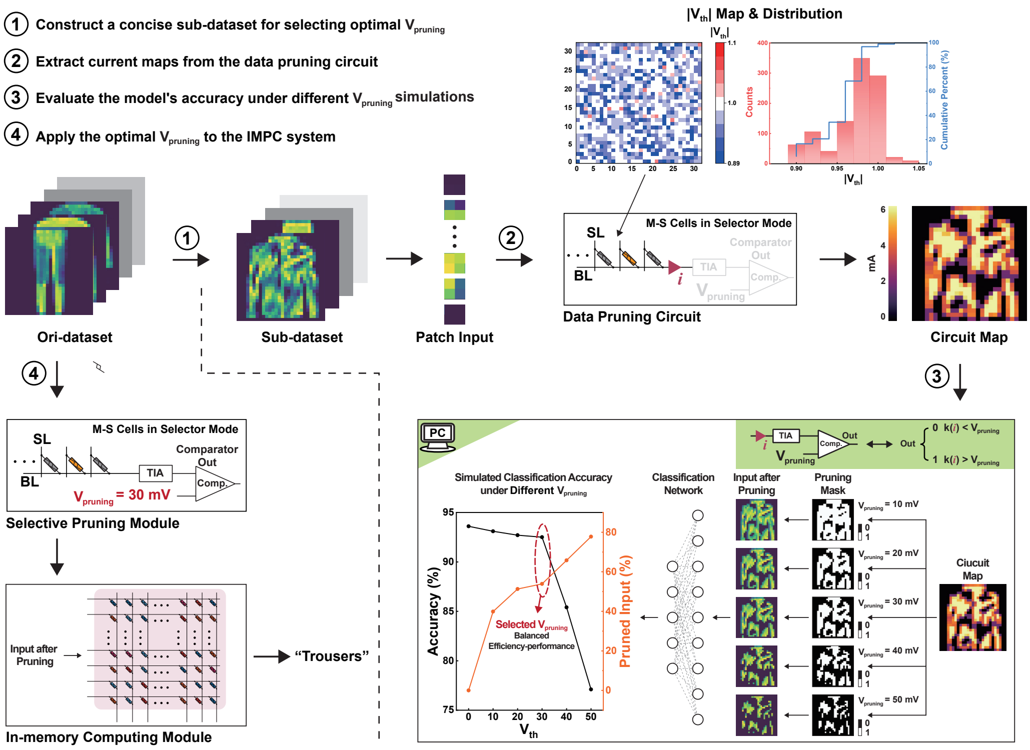


**Figure S24:** Selection method for $V_{pruning}$.

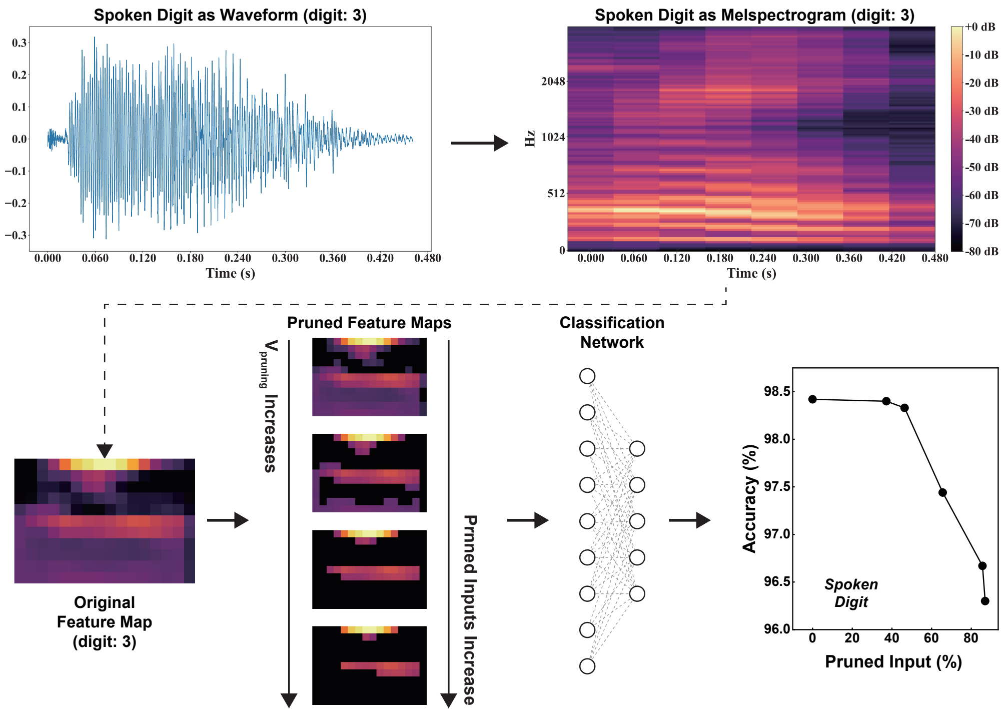


**Figure S25:** Audio classification of the Spoken Digit dataset using the IMPC system.

# S2. Supplementary Tables

Table 1: Resistance state ranges of memristor-mode and selector-mode.

| | Low resistance state | High resistance state | Response time |
|---|---|---|---|
| Memristor-mode | 30 KΩ | 0.2 MΩ | 10 ns |
| | On-state | Off-state | Switch time |
| Selector-mode | 400 Ω | 0.1 MΩ | 20 ns |

Table 2: Hyperparameters and results on the three image classification tasks.

| | MNIST | FashionMNIST | CIFAR-10 |
|---|---|---|---|
| Depth | 2 | 4 | 8 |
| Hidden dimension | 128 | 256 | 256 |
| Patch kernel size | $2 \times 2$ | | |
| Depth kernel size | $5 \times 5$ | | |
| $V_{pruning}$ | 60 mV | 30 mV | 60 mV |
| Input skip | 89.5% | 54.1% | 29.3% |
| Accuracy | 97.0% | 92.7% | 92.6% |

Table 3: Hardware parameters

| Parameter | Value (J) |
|---|---|
| $E_{Driver}$ | 0.17e-15 |
| $E_{Decoder}$ | 0.42e-15 |
| $E_{Mux}$ | 14.06e-15 |
| $E_{ADC}$ | 11.3e-12 |
| $E_{DAC}$ | 0.22e-12 |
| $E_{TIA}$ | 0.27e-12 |
| $E_{Shift\&Adder}$ | 0.37e-12 |
| $E_{Memristor\_Mode}$ | 1.5e-14 |
| $E_{Selector\_Mode\_On}$ | 4.05e-11 |
| $E_{Selector\_Mode\_Off}$ | 9e-15 |

Table 4: Compare with other state-of-the-art work based on devices with threshold and non-volatile resistance switching characteristics. [5, 6, 7, 8]

| | | NC'20 [5] | NC'21 [6] | Science'22 [7] | NC'23 [8] | Ours |
|---|---|---|---|---|---|---|
| Device | **Device Structure** | Pt/Ti/$NbO_x$/ Pt/Ti+Pt/Ta/ $Ta_2O_5$/Pt | Te/$Sb_2Te_3$/Te | Au/$NdNiO_3$/ $LaAlO_3$/Pd | BFO(15Pa)/SRO/ STO+ BFO(19Pa)/ SRO/STO | **TiN/ $Ta_2O_5$/$TaO_x$/Ta TaN** |
| | **Reconfigurable** | No | Yes | Yes | No | **Yes** |
| | **CMOS Compatible** | No | No | No | No | **Yes** |
| | **Crossbar Size** | 4×4 | 2×2 | < 10 | < 10 | **32×32** |
| | **Demonstration Level** | Array | Cell | Cell | Circuit | **Circuit** |
| | **Non-volatile Mode Speed** | 1 µs/1 µs | 10 µs/10 µs | 1 µs | 0.05 s/0.1 s | **30 ns/90 ns** |
| | **Non-volatile Retention** | $> 10^9$ | $> 10^4$ | $> 10^{11}$ | $> 10^6$ | **$> 10^{11}$** |
| | **Volatile Mode Speed** | < 50 ns | 1 ms | 1µs | 2 ms | **20 ns** |
| | **Volatile Endurance** | $> 10^9$ | $> 10^4$ | $> 1.6\times10^6$ | $> 10^6$ | **$> 2.7\times10^{10}$** |

# References


[1] Heeyoung Jeon, Jingyu Park, Woochool Jang, Hyunjung Kim, Chunho Kang, Hyoseok Song, Honggi Kim, Hyungtak Seo, and Hyeongtag Jeon. Stabilized resistive switching behaviors of a pt/taox/tin rram under different oxygen contents. *physica status solidi (a)*, 211(9):2189–2194, 2014.

[2] Hongyang He, Yunlong Gao, Tiejun Li, Yuxiang Lin, Qiao Huang, Ruotong He, Jing Li, Yan Liu, and Jinyan Pan. The shunt conductive effect of ag doped rram via a qualitative circuit model. *Applied Physics A*, 130(10):761, 2024.

[3] Jiao Bai, Weiwei Xie, Weiqi Zhang, Zhipeng Yin, Shengsheng Wei, Dehao Qu, Yue Li, Fuwen Qin, Dayu Zhou, and Dejun Wang. Conduction mechanism and impedance analysis of hfox-based rram at different resistive states. *Applied Surface Science*, 600:154084, 2022.

[4] Shaocong Wang, Yi Li, Dingchen Wang, Woyu Zhang, Xi Chen, Danian Dong, Songqi Wang, Xumeng Zhang, Peng Lin, Claudio Gallicchio, et al. Echo state graph neural networks with analogue random resistive memory arrays. *Nature Machine Intelligence*, 5(2):104–113, 2023.

[5] Qingxi Duan, Zhaokun Jing, Xiaolong Zou, Yanghao Wang, Ke Yang, Teng Zhang, Si Wu, Ru Huang, and Yuchao Yang. Spiking neurons with spatiotemporal dynamics and gain modulation for monolithically integrated memristive neural networks. *Nature communications*, 11(1):3399, 2020.

[6] Yifei Yang, Mingkun Xu, Shujing Jia, Bolun Wang, Lujie Xu, Xinxin Wang, Huan Liu, Yuanshuang Liu, Yuzheng Guo, Lidan Wang, et al. A new opportunity for the emerging tellurium semiconductor: making resistive switching devices. *Nature Communications*, 12(1):6081, 2021.

[7] Hai-Tian Zhang, Tae Joon Park, ANM Nafiul Islam, Dat SJ Tran, Sukriti Manna, Qi Wang, Sandip Mondal, Haoming Yu, Suvo Banik, Shaobo Cheng, et al. Reconfigurable perovskite nickelate electronics for artificial intelligence. *Science*, 375(6580):533–539, 2022.

[8] Zhiwei Chen, Wenjie Li, Zhen Fan, Shuai Dong, Yihong Chen, Minghui Qin, Min Zeng, Xubing Lu, Guofu Zhou, Xingsen Gao, et al. All-ferroelectric implementation of reservoir computing. *Nature communications*, 14(1):3585, 2023.
